# Rapalog resistance is associated with mesenchymal-type changes in Tsc2-null cells

**DOI:** 10.1038/s41598-019-39418-5

**Published:** 2019-02-28

**Authors:** Matthildi Valianou, Natalia Filippidou, Daniel L. Johnson, Peter Vogel, Erik Y. Zhang, Xiaolei Liu, Yiyang Lu, Jane J. Yu, John J. Bissler, Aristotelis Astrinidis

**Affiliations:** 1 Division of Pediatric Nephrology, Department of Pediatrics, College of Medicine, University of Tennessee Health Sciences Center, Memphis, TN 38103 USA; 2 Tuberous Sclerosis Complex Center of Excellence, Le Bonheur Children’s Hospital, Memphis, TN 38103 USA; 3 Office of Research Molecular Bioinformatics Core, University of Tennessee Health Sciences Center, Memphis, TN 38103 USA; 4 Department of Pathology, St Jude Children’s Research Hospital, Memphis, TN 38105 USA; 5 Division of Pulmonary, Critical Care and Sleep Medicine, University of Cincinnati College of Medicine, Cincinnati, OH 45267 USA; 6 Department of Pediatric Medicine, St Jude Children’s Research Hospital, Memphis, TN 38105 USA; 7 Department of Immunotherapeutics and Biotechnology, School of Pharmacy, Texas Tech University Health Sciences Center, Abilene, TX 79601 USA

## Abstract

Tuberous Sclerosis Complex (TSC) and Lymphangioleiomyomatosis (LAM) are caused by inactivating mutations in *TSC1* or *TSC2*, leading to mTORC1 hyperactivation. The mTORC1 inhibitors rapamycin and analogs (rapalogs) are approved for treating of TSC and LAM. Due to their cytostatic and not cytocidal action, discontinuation of treatment leads to tumor regrowth and decline in pulmonary function. Therefore, life-long rapalog treatment is proposed for the control of TSC and LAM lesions, which increases the chances for the development of acquired drug resistance. Understanding the signaling perturbations leading to rapalog resistance is critical for the development of better therapeutic strategies. We developed the first Tsc2-null rapamycin-resistant cell line, ELT3-245, which is highly tumorigenic in mice, and refractory to rapamycin treatment. *In vitro* ELT3-245 cells exhibit enhanced anchorage-independent cell survival, resistance to anoikis, and loss of epithelial markers. A key alteration in ELT3-245 is increased β-catenin signaling. We propose that a subset of cells in TSC and LAM lesions have additional signaling aberrations, thus possess the potential to become resistant to rapalogs. Alternatively, when challenged with rapalogs TSC-null cells are reprogrammed to express mesenchymal-like markers. These signaling changes could be further exploited to induce clinically-relevant long-term remissions.

## Introduction

Tuberous Sclerosis Complex (TSC) and sporadic Lymphangioleiomyomatosis (LAM) are tumor suppressor syndromes sharing the same primary genetic and biochemical features; inactivation of the tumor suppressors *TSC1* or *TSC2*, and aberrant regulation of the PI3K/mTOR pathway. TSC affects 1 in 6,000 individuals with no gender, geographic, or racial bias. Sporadic LAM is extremely rare (approximately 1–5 per million) and affects almost exclusively women. Although comprehensive diagnostic criteria have been established for both TSC^1^ and LAM^2,3^, misdiagnosis is common. Pathologically, TSC is characterized by benign hamartomatous outgrowths in various organs. Malignancies are not common in TSC, and their frequency seems to be similar to that in the general population^4,5^. Neuropsychiatric, renal, and pulmonary manifestations are the main contributors of morbidity and/or mortality in TSC^6–8^. LAM is characterized by diffuse infiltration of the lung parenchyma by smooth muscle-like cells, leading to cystic degeneration and lung destruction. Lung transplantation is the only known treatment for LAM, although cases of recurrent LAM after transplantation have been reported. Most TSC cases are caused by loss-of-function mutations in *TSC1* or *TSC2*, with a small percentage of patients with no mutations identified. Sporadic LAM is caused primarily by mutations in *TSC2*, with a few reports of *TSC1* mutations^9,10^. The current model for sporadic LAM disease assumes that TSC-null cells migrate to and proliferate in the lungs in an estrogen-dependent manner^11^. Indeed, circulating LAM cells have been identified in the peripheral blood of patients^12^. However, the lineage and site of origin of these cells remains elusive.

*TSC1* and *TSC2* encode hamartin and tuberin, respectively. These proteins, together with TBC1D7^13^, form a functional complex which possesses GTPase-activating protein activity specifically against the small GTPase Rheb. GTP-bound Rheb is essential for the activation of mTORC1 on the lysosomal membrane in the presence of amino-acids^14^. mTORC1 is a rapamycin-sensitive multimeric protein complex consisting primarily of the S/T kinase mechanistic target of rapamycin (mTOR), raptor, mLST8, DEPTOR and PRAS40. Active mTORC1 positively regulates mRNA translation, ribosome biogenesis, protein synthesis, nucleotide and lipid biosynthesis, and glucose metabolism, whereas it inhibits autophagy and protein turnover (reviewed in^15,16^). Inactivation of hamartin/tuberin, as in TSC and LAM, results in the hyperactivation of mTORC1. mTOR forms a second, distinct and partially rapamycin-insensitive multimeric complex consisting of mTOR, rictor, mLST8, DEPTOR, Protor1/2, and mSin1. mTORC2 is essential for the full activation of AKT, via direct phosphorylation at residue S473. Other proteins downstream of mTORC2 include PKCγ, SGK and FoxO1/3, which regulate the cytoskeleton and cell migration, ion transport and apoptosis. mTORC2 does not seem to be regulated by the hamartin/tuberin complex or by Rheb. However, inactivation of hamartin/tuberin leads to concomitant loss of mTORC2 activity due to p70S6K-mediated inhibition of rictor^17,18^.

The hamartin/tuberin complex is regulated by direct phosphorylation from a plethora of kinases, including AKT, ERK1/2, RSK1, MK2, AMPK, GSK3, IKKβ, CDK1, and PLK1^19,20^. These phosphorylation events are critical for the integration of signals which lead to the regulation of cell growth through mTORC1 and emphasize the redundancy of signaling networks (e.g. growth factor stimulation through AKT, ERK, and RSK1). Recently, it was found that hamartin is a client and co-chaperon of Hsp90^21,22^, a protein that facilitates protein folding.

The identification of mTORC1 hyperactivation as the main and most important biochemical event related to TSC and LAM pathogenesis^23,24^, led to the first clinical trials and regulatory approval of the mTORC1 inhibitors sirolimus (rapamycin) and everolimus (RAD001) for the management of brain, renal and pulmonary manifestations in TSC and LAM^25–28^. However, several discoveries point toward the notion that rapamycin and its analogues (collectively rapalogs) are far from perfect pharmaceuticals for TSC and LAM treatment. First, although the inhibition of mTORC1 signaling may cause a reduction in size of solid proliferative lesions, these lesions remain. The clinical significance of a treatment that causes some shrinkage, but does *not* eliminate the tumor, may be of uncertain value. All *in vivo* and *in vitro* studies unequivocally proved that rapalog monotherapy does not induce apoptosis in cells; rapalogs act primarily as cytostatic drugs and inhibit cell growth and proliferation through cell cycle arrest in G1/S. More importantly, rapalogs re-activate the pro-survival molecule AKT through two negative feedback loops both originating from p70S6K^17,29^. Once active, AKT inhibits the pro-apoptotic FoxO transcription factors^30^. In addition, mTORC1 is a well-established inhibitor of autophagy, a cancer cell survival process, through its direct inhibitory phosphorylation of key autophagy proteins (reviewed in^31^). Second, discontinuation of treatment leads to renal tumor re-growth and decline in pulmonary function even close to baseline values within a year after treatment cessation^25,32,33^. Despite these drawbacks, rapalogs remain the only drugs for the treatment of renal, pulmonary, and brain lesions in TSC and LAM. Since treatment cessation leads to tumor regrowth, current regimens consist of life-long rapalog use. Considering the latter, development of acquired drug resistance is a concern.

Here, we report the development and comprehensive characterization of the first tuberin-null rapamycin-resistant cell line. Key features of these cells are the loss of epithelial markers, the acquisition of mesenchymal characteristics, the aberrant activation of signaling pathways in addition to PI3K/mTOR, and the enhanced tumorigenicity and metastatic potential.

## Results

### Generation of rapamycin-resistant ELT3 cells

**Figure 1:**
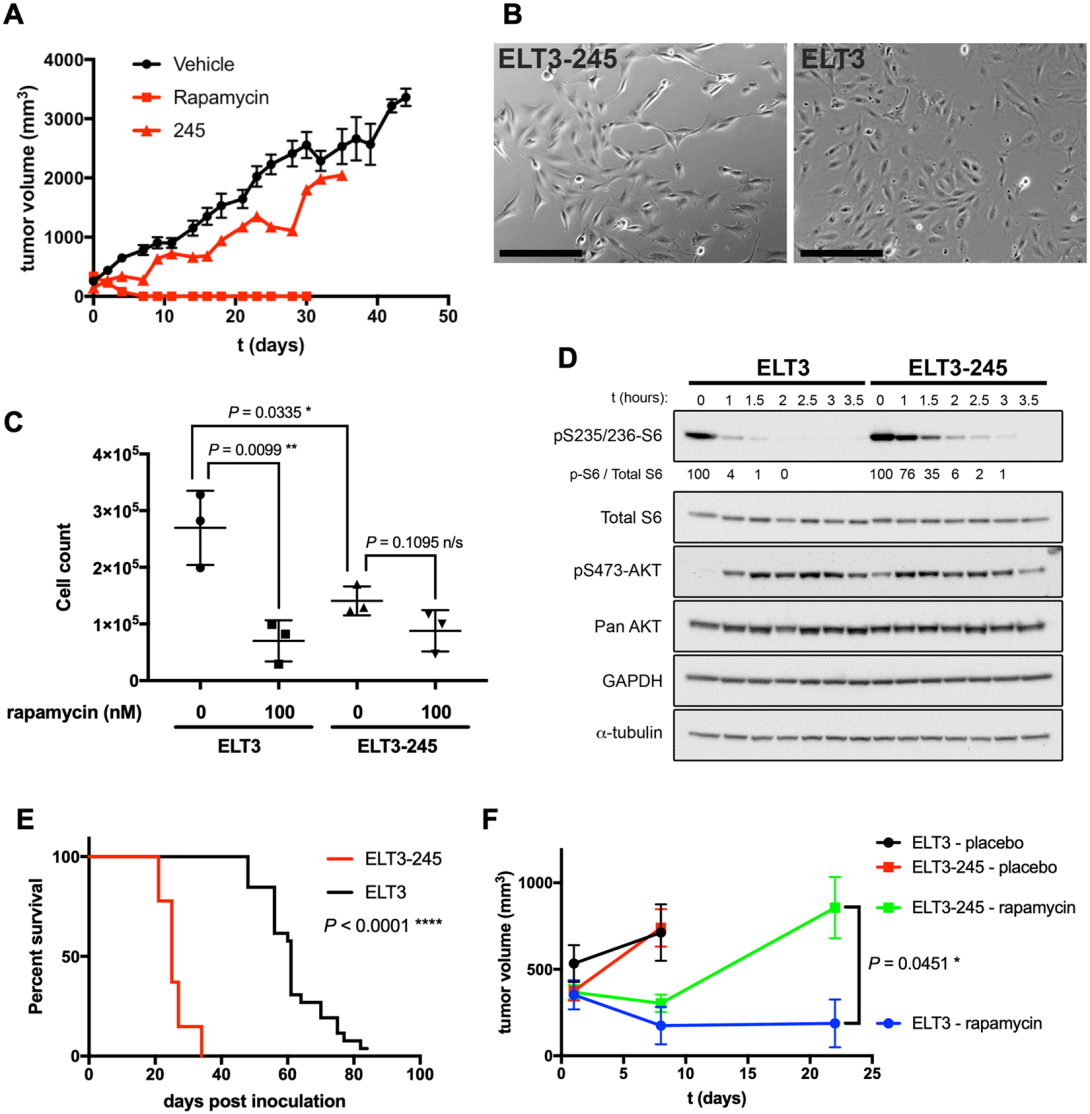
ELT3-245 are resistant to rapamycin. (**A**) Female ovariectomized CB17/SCID mice were inoculated subcutaneously with ELT3 cells. After tumor development, mice were randomized in treatment cohorts of vehicle (*n* = 27, circles, black line), or rapamycin (3 mg/kg ip three times a week, *n* = 19, red lines). A single tumor from mouse #245 in the rapamycin cohort (upward triangles) did not respond to treatment. (**B**) ELT3-245 cells have enhanced spindle-like characteristics, compared to ELT3. Scale is 250 μm. (**C**) Rapamycin has no significant effect on ELT3-245 growth. Equal numbers of ELT3 and ELT3-245 cells (*n* = 3) were cultured for 4 days in the presence of DMSO or rapamycin, and living cells were counted using trypan blue exclusion. (**D**) ELT3-245 cells have delayed dephosphorylation of ribosomal protein S6 upon rapamycin treatment, compared to ELT3. Cells were incubated with 2 nM rapamycin for the indicated time. Ratios of p-S6: Total S6 are normalized to the 0 h time point value within each group. Full-length blots are presented in Supplementary Fig. 3. (**E**) Mice inoculated with ELT3-245 cells develop tumors faster, compared to ELT3. Non-ovariectomized female CB17/SCID mice were inoculated subcutaneously with equal number of ELT3 or ELT3-245 cells (*n* = 26 per group). ELT3 and ELT3-245 tumor-bearing mice were monitored daily for formation of palpable tumors. (**F**) ELT3-245 tumors do not respond to rapamycin treatment *in vivo*. ELT3-245 tumors partially, but not statistically significantly, responded to rapamycin at day 8, however they significantly grew beyond baseline by day 22. ELT3 tumors rapidly responded to rapamycin treatment by day 8, and the response was sustained until day 22. Vehicle-treated tumors exceeded 4,000 mm^3^ before day 22, and mice that reached end-point criteria were removed from the study and euthanized.

Tuberin-null uterine leiomyoma cells (ELT3) derived from an Eker rat are tumorigenic in immunodeficient mice^34^. During the course of ELT3 xenograft studies in CB17/SCID mice, we identified one mouse (#245) bearing a tumor that did not respond to rapamycin treatment (Fig. 1A). Rapamycin plasma concentration was 25 ng/ml three days after final treatment, higher than human therapeutic trough levels (4–20 ng/ml). The tumor was explanted under aseptic conditions, tumor cells were dissociated and used to establish a cell line, termed ELT3-245. The rat origin of ELT3-245 cells and the absence of contaminating mouse cells was confirmed by qPCR using rat- and mouse-specific primers and probes^34^ (data not shown). Morphologically, ELT3-245 cells are more spindle-like (Fig. 1B), compared to parental ELT3 that resemble an epithelioid leiomyoma cell line as originally described^35^. Since mutations in the FKBP12-rapamycin binding (FRB) domain of mTOR are associated with rapamycin resistance in cancer^36^, we performed direct sequencing of exons 41–46 of rat *Frap1* (mTOR), which correspond to the FRB domain. We did not find any mutations in the FRB domain of ELT3-245, compared to ELT3 and to the rat reference genome (data not shown). To validate that ELT3-245 cells are rapamycin-resistant, we studied their response to rapamycin treatment *in vitro* (Figs 1C and S1). Rapamycin treatment of ELT3-245 slowed down but not significantly inhibited their growth over 4 days (0 nM rapamycin: 140,667 ± 14,769, *n* = 3; 100 nM rapamycin: 87,867 ± 21,083, *n* = 3; *P* = 0.1095), contrasting the strong rapamycin-induced inhibition of parental ELT3 cells (0 nM rapamycin: 269,667 ± 37,746, *n* = 3; 100 nM rapamycin: 70,167 ± 21,014, *n* = 3; *P* = 0.0099). Untreated ELT3-245 cells had significantly slower growth rate, compared to parental ELT3 cells (ELT3: 269,667 ± 37,746, *n* = 3; ELT3-245: 140,667 ± 14,769, *n* = 3; *P* = 0.0335), despite the slightly increased levels of PCNA (Fig. S2). Treatment of ELT3-245 cells with 0.2 nM rapamycin for 24 hours led to a reduction of ribosomal protein S6 phosphorylation, but to a lesser extent compared to parental ELT3 (Fig. S2). A time-course study revealed that, upon rapamycin treatment, ELT3-245 cells show significantly delayed dephosphorylation of S6, compared to ELT3 (Figs 1D and S3). Of note, untreated ELT3-245 cells had increased phosphorylation of AKT at S473, a mTORC2 phosphorylation site, compared to parental ELT3 cells.

Next, we addressed whether ELT3-245 cells are tumorigenic and if they respond to rapamycin *in vivo*. CB17/SCID mice were inoculated bilaterally with equal numbers of ELT3 or ELT3-245 cells and assessed daily for palpable tumors. ELT3-245 cells formed tumors earlier than expected, compared to parental ELT3 cells (Fig. 1E); the tumor-free survival for ELT3-245 and ELT3 was 25 and 61 days, respectively (Log-rank Mantel-Cox and Gehan-Breslow-Wilcoxon *P* < 0.0001). When tumors reached an average volume of ≈400 mm^3^ (ELT3: 472.9 mm^3^ ± 78.91, n = 9; ELT3-245: 370.1 mm^3^ ± 31.93, *n* = 24; *P* = 0.1554), mice were randomized into treatment groups and received either placebo (vehicle ip, 3 times per week) or rapamycin (3 mg/kg ip, 3 times per week). Upon treatment with rapamycin, ELT3-245 tumors partially, but not statistically significantly, responded to treatment at early time points (Figs 1F and S4A); however, by day 22 post-treatment ELT3-245 tumors were significantly larger compared to day 1 (*P* < 0.01, Fig. S4A). More importantly, at day 22 rapamycin-treated ELT3-245 tumors were significantly larger compared to ELT3 tumors (Figs 1F and S4B). Taken together, these data support that ELT3-245 is a truly rapamycin-resistant cell line derived from Tsc2-null precursors.

### Tumorigenic characteristics of ELT3-245

**Figure 2:**
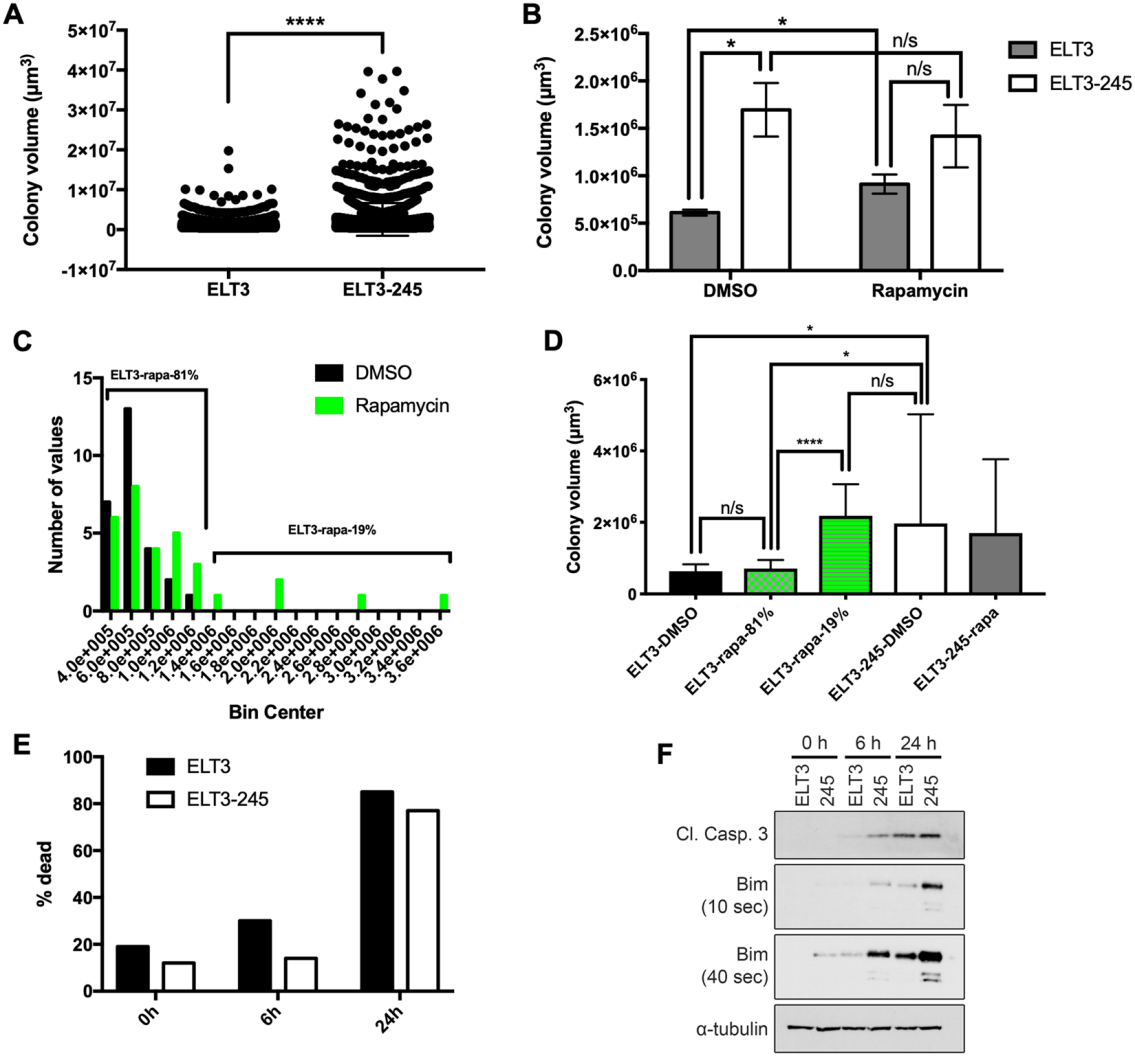
ELT3-245 cells exhibit enhanced anchorage-independent cell growth and resistance to anoikis. (**A**) Automatic particle analysis of colonies from soft agar assay. ELT3-245 cells formed significantly bigger colonies, compared to ELT3 cells (****P < 0.0001). (**B**) 24 hours after plating ELT3 and ELT3-245 cells in soft agar, cultures were continuously treated with 20 nM rapamycin for 3 weeks. Rapamycin treatment did not cause a significant reduction in colony volume in ELT3-245 cells, compared to DMSO. However, rapamycin caused a significant *increase* in colony volume in ELT3 cells (*P = 0.0148). (**C**) A subset of ELT3 cells are rapamycin-resistant. Volume distribution of DMSO- and rapamycin-treated parental ELT3 in soft agar. Approximately 19% of rapamycin-treated ELT3 colonies were bigger than DMSO-treated ELT3 colonies. (**D**) The top 19% of rapamycin-treated ELT3 colonies were significantly bigger, compared to the bottom 81% of rapamycin-treated ELT3 colonies (*P* < 0.0001). The top 19% of rapamycin-treated ELT3 colonies were *not* significantly bigger, compared to DMSO-treated ELT3-245 colonies (*P* > 0.05). (**E**) ELT3 and ELT3-245 cells were grown in non-adherent condition for 6 and 24 hours, harvested and the percentage of dead cells was counted by trypan blue exclusion. 0 h indicates percentage of dead cells immediately after trypsinization and resuspension in growth media. (**F**) Immunoblotting of lysates from cells grown in suspension conditions for 0, 6 and 24 hours from panel E. Full-length blots are presented in Supplementary Fig. 6.

Our xenograft study showed that ELT3-245 have increased tumorigenicity (Fig. 1E), compared to parental ELT3 cells. To explore the potential mechanisms implicated in this phenomenon we conducted anchorage-independent cell growth (soft agar) and cell death (anoikis) assays. First, single-cell suspensions of ELT3 and ELT3-245 cells were embedded in agarose and allowed to form colonies for 2–3 weeks. Particle size analyses revealed that ELT3-245 colonies were significantly bigger than parental ELT3 colonies (ELT3: 1,178,043 μm^3^ ± 24,139, *n* = 2,105; ELT3-245: 2,568,395 μm^3^ ± 92,107, *n* = 1,994; *P* < 0.0001) (Figs 2A and S5). Consistent with ELT3-245 cells being rapamycin-resistant, rapamycin treatment did not cause a significant reduction in colony volume of ELT3-245 cells, compared to cells treated with DMSO (DMSO: 1,968,417 μm^3^ ± 388,787, *n* = 62; rapamycin: 1,699,120 μm^3^ ± 421,817, *n* = 24; *P* > 0.05) (Fig. 2B). Unexpectedly, rapamycin induced a significant *increase* in colony volume of ELT3 cells (DMSO: 635,435 μm^3^ ± 38,104, *n* = 27; rapamycin: 997,478 μm^3^ ± 129,980, *n* = 31; *P* = 0.0148). We analyzed the volume distribution of DMSO- and rapamycin-treated ELT3 colonies and found that 19% of rapamycin-treated ELT3 colonies were bigger than the maximum volume of DMSO-treated ELT3 colonies (Fig. 2C). This top 19% of rapamycin-treated ELT3 colonies were significantly bigger, compared to the bottom 81% of rapamycin-treated ELT3 colonies (ELT3-rapa-19%: 2,181,332 μm^3^ ± 361,144, *n* = 6; ELT3-rapa-81%: 713,353 μm^3^ ± 47,823, *n* = 25; *P* < 0.0001) (Fig. 2D). Most importantly, the top 19% of rapamycin-treated ELT3 colonies did not have significantly different volume, compared to DMSO-treated ELT3-245 (ELT3-rapa-19%: 2,181,332 μm^3^ ± 361,144, *n* = 6; ELT3-245-DMSO: 1,968,417 μm^3^ ± 388,787, *n* = 62; *P* = 0.8666). These data suggest that a subset of ELT3 cells are rapamycin-resistant.

Cancer cells are resistant to anchorage-independent cell death (anoikis). Under detached conditions, ELT3 cells undergo anoikis, while estradiol increases their resistance to anoikis^34^. Since ELT3-245 are more tumorigenic than parental ELT3, we hypothesized that ELT3-245 may be more resistant to anoikis. Parental ELT3 had significantly higher fraction of dead cells at 0 and 6 hours under detached conditions compared to ELT3-245 (ELT3: 0 h = 20%, 6 h = 30%; ELT3-245: 0 h 15%, 6 h 17%) (Fig. 2E). At 24 hours under detached conditions, the difference on cell death between the two cell lines was diminished. Unexpectedly, immunoblotting of lysates from cells under detached conditions showed that ELT3-245 cells have *increased* cleaved caspase 3 and Bim, a pro-apoptotic Bcl-2 family member, compared to parental ELT3 cells (Figs 2F and S6).

### Lung colonization of ELT3-245 cells

**Figure 3:**
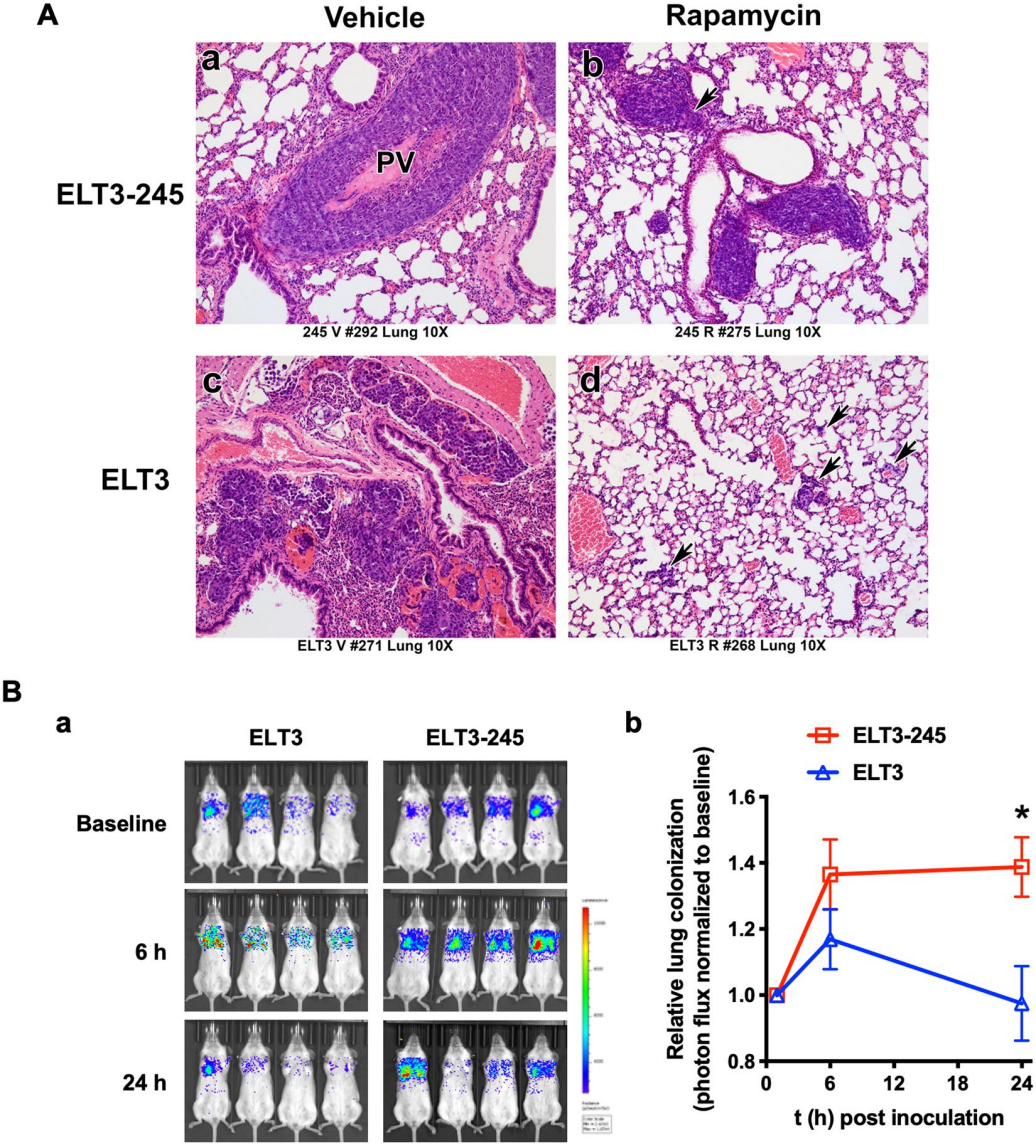
ELT3-245 cells metastasize to the lungs. (**A**) Micrographs (10x objective) of hematoxylin-eosin stained sections from lungs of ELT3-245 (a,b) and ELT3 (c,d) tumor-bearing mice treated with vehicle (a,c) or rapamycin (b,d). PV = pulmonary vein. Arrow in panel b indicates invasion of ELT3-245 cells through basal membrane. Arrows in panel d indicate the location of ELT3 micro-metastases. (**B**) a Bioluminescence images of vehicle-treated SCID mice that were inoculated with 2 × 10^5^ luciferase-expressing ELT3 or ELT3-245 cells pre-treated with DMSO for 16 hours. Mice (n = 4 per group) were imaged at 1 h (baseline), 6 h and 24 h post-inoculation. b Plot of the relative lung colonization (photon flux normalized to baseline) for the mice shown in panel a. *Indicates *P* < 0.05. Error bars are SEM.

Given the increased *in vitro* and *in vivo* tumorigenic potential of ELT3-245 and resistance to anoikis, we examined the lungs of ELT3-245 tumor-bearing mice for metastases. ELT3 cells are known to metastasize into and colonize the lungs of estradiol-supplemented SCID mice^34^. These metastases are usually microscopic, consisting of 10–30 cells, and are dispersed throughout the lung. In ELT3-245 tumor-bearing mice, we observed macroscopic lesions localized primarily in the vascular spaces surrounding the lungs (e.g. pulmonary veins, lymphatics) and the mediastinum (Fig. 3A, panel a), compared to ELT3 cells that localize to the lung parenchyma and alveolar space (Fig. 3A, panel c). Micro-metastasis (small groups of cells) were absent from the lungs of ELT3-245 tumor-bearing mice, but were common in the lungs of ELT3 tumor-bearing mice. Overall, ELT3-245 tumor-bearing mice had increased tumor burden in the lungs, compared to ELT3. ELT3-245 cells are more tightly packed and homogeneous, compared to ELT3 that are more dispersed and heterogeneous with some very anaplastic cells (Fig. S7). Rapamycin almost completely eliminated the hypertrophic and anaplastic cells found in the lung metastases of ELT3 cells (Fig. S7), but had no noticeable effect in the lung tumor burden (Fig. 3A, panel b) or morphology of ELT3-245 cells (Fig. S7). Interestingly, in one mouse treated with rapamycin, ELT3-245 cells seem to invade through the perivascular basal membrane into the lung parenchyma and develop metastases (Fig. 3A, panel b).

Since ELT3-245 cells have a much shorter latency in tumor formation, compared to ELT3 (Fig. 1E), the differences in lung colonization between ELT3 and ELT3-245 cells could reflect differences in the duration of shedding from primary tumor sites. Therefore, we sought to determine the ability of ELT3-245 cells to colonize the lungs immediately after tail-vein injection in SCID mice. Using live bioluminescence imaging, we compared the lung-colonizing capacity between luciferase-expressing ELT3-245 and ELT3 cells^34^, and found that significantly more ELT3-245 cells colonized mouse lungs 24 hours post-inoculation, compared to ELT3 cells (normalized photon flux at 24 h post cell inoculation: ELT3: 0.9747 ± 0.1126, *n* = 4; ELT3-245: 1.3879 ± 0.0904, *n* = 3; *P* = 0.0430; Fig. 3B). Despite ELT3-245 primary tumors being refractory to rapamycin treatment (Fig. 1F), rapamycin significantly reduced the short-term lung colonization by ELT3-245 cells (Fig. S8).

### Mesenchymal characteristics of ELT3-245 cells

**Figure 4:**
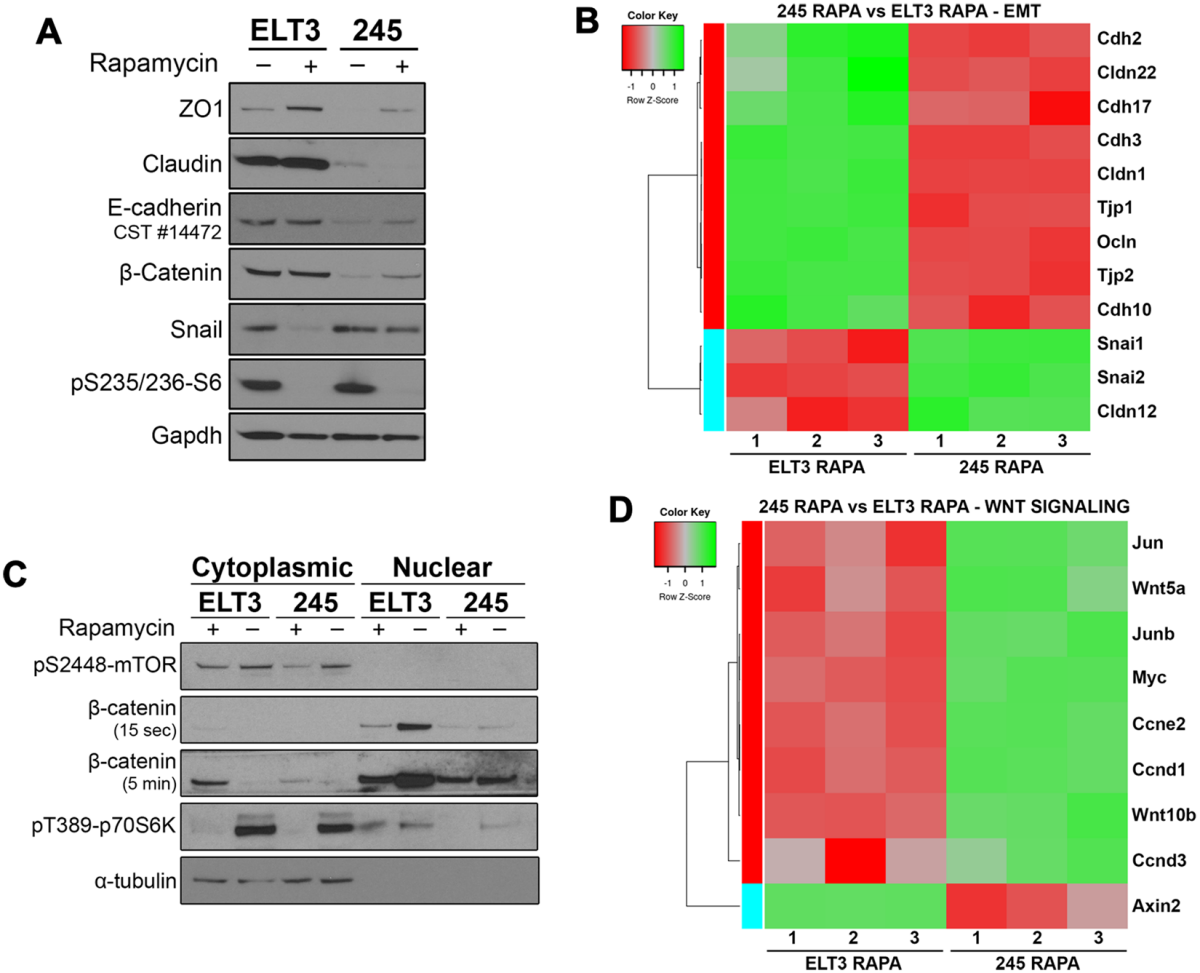
ELT3-245 cells exhibit mesenchymal signaling characteristics. (**A**) EMT marker immunoblotting of ELT3 and ELT3-245 lysates from untreated cells or cells treated with 100 nM rapamycin for 3 days. Full-length blots are presented in Supplementary Fig. 9. (**B**) Heat map of epithelial marker associated genes that were differentially expressed between rapamycin treated ELT3-245, compared to rapamycin treated ELT3. (**C**) Nuclear and cytoplasmic fractions of ELT3 and ELT3-245 cells treated with 100 nM rapamycin (or DMSO) for 3 days were immunoblotted for β-catenin. Full-length blots are presented in Supplementary Fig. 10. (**D**) Heat map of β-catenin target and Wnt signaling genes that were differentially expressed between rapamycin treated ELT3-245, compared to rapamycin treated ELT3.

Since rapalog resistance has been previously associated with cancer cells undergoing epithelial-to-mesenchymal transition (EMT)^37^, we screened for known epithelial and mesenchymal markers in ELT3 and ELT3-245 cells. Compared to the ELT3 parental cell line, ELT3-245 cells exhibited loss of the tight junctions proteins ZO-1 and Claudin 1, and a decrease in the adherens junctions proteins E-cadherin and β-catenin (Figs 4A, S9 and S2). N-cadherin, a mesenchymal marker, was not detected in ELT3-245 or ELT3 cell lysates. In ELT3-245 cells, rapamycin treatment partially restored the protein levels of ZO-1, β-catenin and E-cadherin, but not Claudin 1. Importantly, in ELT3-245 cells rapamycin failed to decrease Snail, a transcriptional repressor of E-cadherin, consistent with the partial restoration of β-catenin and E-cadherin.

In agreement with the decreased protein levels of Claudin 1 in ELT3-245 lysates, a gene expression array study showed that *Cldn1* expression was 33-fold down-regulated in rapamycin-treated ELT3-245, compared to rapamycin-treated ELT3 (Table 1, Fig. 4B, and Supplementary Data 1). Similarly, expression of *Tjp1* and *Tjp2* (encoding ZO-1 and ZO-2, respectively) was more than 2.7-fold down-regulated in ELT3-245 cells, compared to parental ELT3, consistent with the decreased protein levels of ZO-1. Expression of *Ocln* (Occludin) was down-regulated 139-fold in rapamycin-treated ELT3-245, compared to rapamycin-treated ELT3, and down-regulated 35-fold in non-treated ELT3-245, compared to non-treated ELT3. *Snai1* (Snail) and *Snai2* (Slug) was up-regulated by approximately 3- and 6-fold respectively in rapamycin-treated and non-treated ELT3-245, compared to ELT3. Expression of *Cdh1* (E-cadherin) was not differentially regulated between ELT3-245 and ELT3 cells, both under rapamycin treatment and no treatment conditions. Interestingly, *Cdh2* (N-cadherin) was down-regulated in ELT3-245 vs ELT3, consistent with our inability to detect N-cadherin in cell lysates using at least two different antibodies (data not shown). Several other cadherin-expressing genes (*Cdh3*, *Cdh10*, *Cdh17*) were also down-regulated in ELT3-245, compared to ELT3. Taken together, these data confirm that ELT3-245 cells have loss of multiple tight junctions and adherens junctions proteins.

**Table 1:** Expression of EMT genes (A), β-catenin target genes and Wnt/β-catenin signaling pathway genes (B), and ErbB signaling, ECM-receptor interaction signaling and VEGF genes.

Gene	Protein		DMSO			Rapamycin		
			FC	Regulation	*P*	FC	Regulation	*P*
**(A) EMT genes**								
***Snai1***	**Snail**		**2.48**	**UP**	**0.0004**	**3.06**	**UP**	**0.0053**
***Snai2***	**Slug**		**6.87**	**UP**	**1.35E-05**	**6.24**	**UP**	**1.00E-05**
***Ocln***	**Occludin**		**34.83**	**DOWN**	**0.0022**	**139.31**	**DOWN**	**3.02E-05**
***Tjp1***	**ZO-1**		**2.70**	**DOWN**	**0.0004**	**3.69**	**DOWN**	**0.0007**
***Tjp2***	**ZO-2**		**3.85**	**DOWN**	**0.0002**	**3.73**	**DOWN**	**0.0002**
***Cldn1***	**Claudin 1**		**23.75**	**DOWN**	**4.69E-07**	**32.80**	**DOWN**	**0.0003**
***Cldn12***	**Claudin 12**		**1.83**	**UP**	**0.0204**	**2.18**	**UP**	**0.0067**
*Cldn22*	Claudin 22		1.11	DOWN	n/s	**1.50**	**DOWN**	**0.0384**
*Cdh1*	E-cadherin		1.53	UP	n/s	1.04	DOWN	n/s
***Cdh2***	**N-Cadherin**		**2.67**	**DOWN**	**0.0068**	**4.61**	**DOWN**	**0.0157**
***Cdh3***	**P-cadherin**		**11.81**	**DOWN**	**4.14E-06**	**9.36**	**DOWN**	**6.39E-06**
***Cdh10***	**Cadherin 10**		**2.12**	**DOWN**	**0.0102**	**2.75**	**DOWN**	**0.0004**
***Cdh17***	**Cadherin 17**		**5.06**	**DOWN**	**3.69E-05**	**3.71**	**DOWN**	**0.0042**
**(B) β-catenin target genes and Wnt/β-catenin signaling pathway genes**								
***Axin2***	**Axin 2**		**5.32**	**DOWN**	**0.0017**	**2.39**	**DOWN**	**0.0271**
***Jun***	**c-jun**		**3.35**	**UP**	**0.0030**	**3.99**	**UP**	**0.0110**
***Junb***	**Jun B Proto-Oncogene (AP-1 transcription factor)**		**10.88**	**UP**	**0.0002**	**13.64**	**UP**	**0.0007**
***Myc***	**c-myc**		**1.74**	**UP**	**0.0121**	**2.55**	**UP**	**0.0003**
*Ccnd1*	Cyclin D1		1.10	UP	n/s	**1.75**	**UP**	**0.0007**
*Ccnd3*	Cyclin D3		1.25	UP	0.0017	**1.69**	**UP**	**0.0019**
*Ccne1*	Cyclin E1		**1.75**	**DOWN**	**0.0073**	1.19	DOWN	0.0252
*Ccne2*	Cyclin E2		1.03	UP	n/s	**1.59**	**UP**	**0.0008**
*Wnt10b*	Wnt Family Member 10B		1.30	UP	n/s	**2.21**	**UP**	**0.0003**
*Wnt5a*	Wnt Family Member 5 A		1.99	UP	n/s	**1.88**	**UP**	**0.0049**
**(C) ErbB and ECM-receptor interaction signaling pathways genes**								
*Egfr*		Epidermal Growth Factor Receptor	1.456	UP	n/s	**2.13**	**UP**	**0.0246**
*Map2k2*		MAP kinase kinase 2	1.04	UP	n/s	**1.55**	**DOWN**	**0.0072**
*Mapk3*		p44 MAPK	1.44	DOWN	0.0075	**2.29**	**DOWN**	**0.0001**
*Pik3r3*		PI3K regulatory subunit γ	1.34	UP	n/s	**1.87**	**DOWN**	**0.0052**
*Akt2*		AKT Serine/Threonine Kinase 2	1.38	UP	0.0188	**1.52**	**UP**	**0.0048**
*Akt3*		AKT Serine/Threonine Kinase 3	2.02	DOWN	n/s	**1.95**	**DOWN**	**0.0031**
*Itgav*		Integrin αV	1.46	DOWN	0.0158	**1.94**	**DOWN**	**0.0005**
***Itgb3***		**Integrin β3**	**3.57**	**DOWN**	**0.0090**	**5.10**	**DOWN**	**0.0005**
*Itgb5*		Integrin β5	1.44	DOWN	2.12E-05	**1.68**	**DOWN**	**0.0009**

Genes that are differentially regulated under both control (DMSO) and rapamycin (20 nM for 24 hours) conditions are shown in bold. FC is the fold-change in ELT3-245 vs ELT3 cells. The *P* value was calculated from gene expression data of triplicate samples. n/s indicates *P* ≥ 0.05. Complete gene expression data can be found in Supplemental Information.

β-catenin is regulated by GSK3β-dependent phosphorylation, which targets it for ubiquitination and eventual proteasomal degradation. When the Wnt/Frizzled/GSK3β signaling pathway is activated, β-catenin is stabilized and translocated to the nucleus where it complexes with additional transcriptional factors to regulate gene expression. To examine the translocation of β-catenin in the nucleus, we performed nuclear and cytoplasmic fractionation from ELT3 and ELT3-245 cells, in the absence and presence of rapamycin. Rapamycin decreased β-catenin in the nuclear fraction of ELT3 cells, compared to control-treated cells, and increased β-catenin in the cytoplasmic fraction (Figs 4C and S10), suggesting that rapamycin prevents β-catenin nuclear translocation and/or promotes its cytoplasmic retention. In contrast to ELT3, in ELT3-245 cells β-catenin nuclear levels did *not* change in the presence of rapamycin. Several β-catenin target genes were up-regulated in ELT3-245 cells in our gene expression array data, including *Jun* (c-jun), *Junb* (JunB proto-oncogene, or transcription factor AP-1), and *Myc* (c-myc), both in no treatment and rapamycin treatment conditions (Table 1, Fig. 4D, and Supplementary Data 2). *Ccnd1*, *Ccnd3*, and *Ccne2* (encoding Cyclins D1, D3 and E2, respectively) were also up-regulated in ELT3-245, compared to parental ELT3, but only under rapamycin treatment conditions. Integrated pathway analysis confirmed differential regulation of the Wnt signaling pathway in rapamycin-treated ELT3-245 cells, compared to parental ELT3 (Fig. S11).

**Figure 5:**
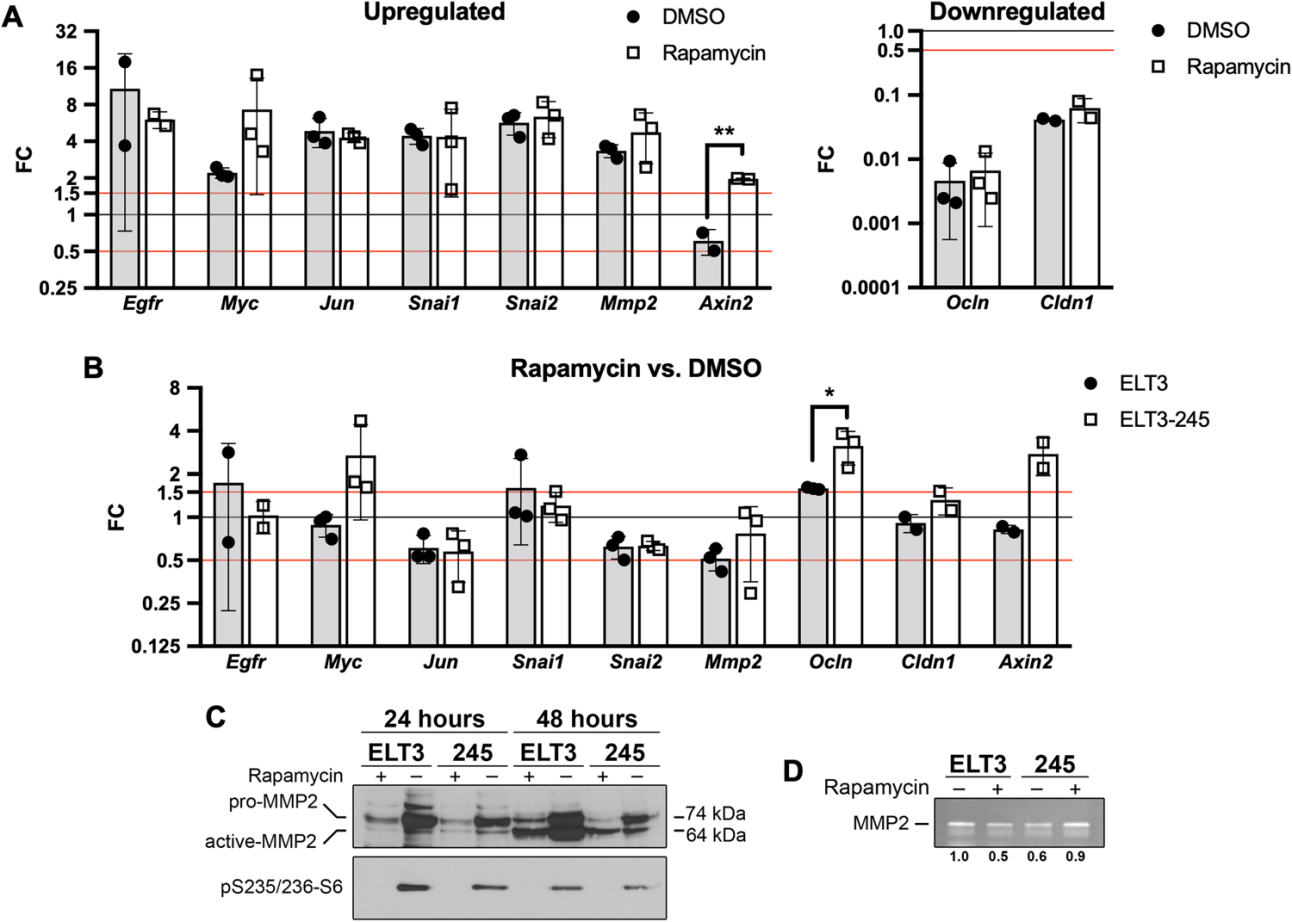
Gene expression changes associated with ELT3-245 cells. (**A**) Gene expression analysis of upregulated (left) and downregulated (right) genes in ELT3-245 cells vs ELT3 cells under vehicle treatment (DMSO, gray bars, closed circles) or rapamycin treatment (20 nM for 24 hours, white bars, open squares). The 0.5 < FC < 1.5 thresholds for significant gene expression differences are indicated with red horizontal lines. (**B**) Effect of rapamycin treatment vs DMSO treatment on gene expression in ELT3 (gray bars, closed circles) and ELT3-245 cells (white bars, open squares). (**C**,**D**) ELT3 and ELT3-245 cells were cultured in serum-free media for 24 or 48 hours, in the presence of 100 nM rapamycin (or DMSO). (**C**) Lysates were immunoblotted for MMP2. Full-length blots are presented in Supplementary Fig. 13. (**D**) Media from cell cultures were collected and analyzed by zymography for MMP2 activity. Numbers below the zymogram gels show relative activity of MMP2 compared to DMSO-treated ELT3 cells. Full-length gel is presented in Supplementary Fig. 14.

To validate the gene expression array findings, we performed independent experiments to assess relative gene expression by RT-qPCR. Expression of several target genes were up-regulated in ELT3-245 cells, compared to ELT3, including *Egfr*, *Myc*, *Jun*, *Snai1*, *Snai2*, and *Mmp2* (Fig. 5A) in both rapamycin-treated and control cultures. *Ocln* and *Cldn1* were down-regulated in ELT3-245 cells, compared to ELT3. These results are in agreement with the gene expression array data (Table 1). *Axin2* expression was significantly upregulated (FC > 1.5) under rapamycin treatment in ELT3-245 cells, compared to ELT3 (Fig. 5A); however, *Axin2* expression was *not* significantly changed (0.5 < FC < 1.5) in control ELT3-245 cells, which contrasted array data. The FC values for *Axin2* between rapamycin- and control-treated cells were statistically significantly different. The FC values for *Myc* expression (ELT3-245 vs ELT3) under rapamycin treatment were higher, but not statistically significantly different, than the corresponding FC values under no-treatment conditions. When studying the effect of rapamycin treatment on expression of these nine genes (Fig. 5B), we observed that rapamycin treatment significantly increased *Myc, Ocln, and Axin2* expression in ELT3-245 cells (FC > 1.5). In ELT3 cells rapamycin had no effect on expression of *Myc* and *Axin2* in ELT3 cells (0.5 < FC < 1.5), and had a marginal effect on expression of *Ocln*.

Increased MMP2 and MMP9 were previously shown to be most relevant with LAM pathology in animal models and human specimens^38,39^. *Mmp2* expression was up-regulated in ELT3-245 cells, compared to ELT3 (Figs 3A, 5A and S12). *Mmp9* was not among the differentially expressed matrix metalloproteinase genes. *Mmp15*, which was also upregulated in ELT3-245 (Fig. S12), was previously reported to be upregulated in Tsc2-null cells in a rapamycin-insensitive manner^40^. We analyzed MMP2 protein levels and activity and found that the active MMP2 (64 kDa) protein levels were increased during 48-hour serum starvation both in ELT3 and ELT3-245 cells (Figs 5C and S13), compared to 24-hour serum starvation. At the 48-hour time-point rapamycin (100 nM) decreased active-MMP2 only in ELT3 cells. Rapamycin treatment had no effect in active-MMP2 levels in the ELT3-245 cells. Next, we studied MMP2 gelatinase activity in serum-free culture media from ELT3 and ELT3-245 cells in the presence or absence of 100 nM rapamycin for 48 hours. Consistent with a decrease of active-MMP2 in ELT3 cells caused by rapamycin (Fig. 5C), MMP2 activity was significantly inhibited in media from rapamycin-treated ELT3 (Figs 5D and S14). In contrast, rapamycin did not decrease MMP2 activity in media from ELT3-245 cultures. In the contrary, MMP2 activity was slightly, but notably, increased in rapamycin-treated ELT3-245. Untreated ELT3-245 cells had lower active-MMP2 protein levels (Fig. 5C) and lower MMP2 gelatinase activity (Fig. 5D) compared to untreated ELT3.

Taken together, these results are in line with a role of β-catenin in the transcriptional regulation of tumorigenic signaling molecules and, most importantly, the differential effect of rapamycin in nuclear β-catenin translocation and activity in the rapamycin-resistant ELT3-245 cells, compared to ELT3.

### ErbB and Extracellular Matrix (ECM)-receptor interaction signaling pathways are differentially regulated in ELT3-245 cells

Analysis for differentially regulated pathways between ELT3-245 and ELT3 cells, showed up-regulation of key signaling pathways contributing to metastasis and tumorigenesis in cancer, including ErbB and ECM-receptor interaction. The ErbB signaling pathway was differentially regulated only in the presence of rapamycin (Table 1, Figs S15 and S16, Supplementary Data 3). The genes involved were *Egfr* (Epidermal Growth Factor Receptor), *Map2k2* (MAP kinase kinase 2), *Mapk3* (p44 MAPK), *Pik3r3* (PI3K regulatory subunit g), *Akt2* (AKT Serine/Threonine Kinase 2) and *Akt3* (AKT Serine/Threonine Kinase 3). For ECM-receptor interaction signaling the up-regulated genes were *Itgav* (integrin αV) and *Itgb5* (integrin β5) (Table 1, Figs S17 and S18), which are implicated in angiogenesis^41–43^, increased vascular permeability, and VEGF production^44,45^.

## Discussion

Despite advances in understanding the genetic and biochemical etiology of TSC and LAM pathogenesis, and the approval and clinical use of rapalogs for the treatment of neurological, renal and pulmonary manifestations, the inherent limitations of these therapeutics make further research for the development of effective therapeutic approaches a pressing matter. Rapalog therapy is quite new, and acquired rapalog resistance in TSC and LAM patients undergoing therapy is a significant risk. Lesions in TSC and LAM are slow-progressing and may take years to develop and become clinically significant, which contrasts the rapidly-growing cases of malignancies (breast cancer, renal cell carcinoma) for which rapalog resistance has been reported. We can predict that any acquired drug resistance in TSC and LAM may emerge after multiple years of treatment. Therefore, there is increasing concern among the medical and scientific community that resistance to rapalogs may slowly emerge and may cause irreversible harm to TSC and LAM patients and limit, or worse eliminate, treatment options. Moreover, tumors from a percentage of TSC and LAM patients are not responsive to rapalog treatment^33^.

Resistance to rapamycin has been previously reported for breast cancer and renal cell carcinoma^36,46^, and various mechanisms have been postulated, including a shift in the balance from the rapamycin-sensitive mTORC1 to the rapamycin-insensitive mTORC2, increased expression of phospholipase D^47^, re-activation of AKT and MAPK signaling pathways, and acquisition of mutations in the FRB and kinase domains of mTOR in a variety of tumors^36^.

During xenograft studies of *Tsc2*-null Eker rat ELT3 cells, which have been used extensively as models for TSC and LAM, we identified a rapamycin non-responsive tumor which was used to develop a rapamycin-resistant cell line, termed ELT3-245. These cells are highly tumorigenic in mice, with evident resistance to rapamycin treatment and increased tumor burden to the lungs. In fact, ELT3-245 cells develop macroscopic metastases within the vascular component of the lungs, as opposed to parental ELT3 which develop primarily micro-metastases in the lung parenchyma and alveolar space^34^. Our short-term lung colonization studies, revealed that ELT3-245 cells colonize the lungs more than ELT3 cells, consistent with increased resistance to anoikis. Interestingly though, rapamycin decreased ELT3-245-induced lung colonization, and this could reflect a differential effect of rapamycin on the capacity of ELT3-245 cells to proliferate and form tumors at the primary site vs their metastatic/migratory potential. Despite of increased levels of apoptotic markers (cleaved caspase 3 and Bim), ELT3-245 cells are resistant to anchorage-independent cell death (anoikis) and form larger colonies in soft agar assays, which are in agreement with their increased *in vivo* tumorigenicity and metastatic potential. ELT3-245 growth rate is slower than the parental ELT3 cells, which could be the net result of increased proliferation rate (evident by increased PCNA protein and increased expression for Cyclin D and E genes) counteracted by increased apoptosis.

EMT has been previously associated with rapamycin resistance in breast cancer cell lines; rapamycin-resistance cells exhibit loss of E-cadherin, and expression of a constitutively active form of Snail decreases response to rapamycin of drug-sensitive cells^37^. ELT3-245 cells have loss of various epithelial markers found in ELT3 cells, either at the gene expression or protein level or both. These include E-cadherin, ZO-1, Occludin, and Claudin 1. Although we could not identify expression of mesenchymal markers, ELT3-245 cells have acquired a mesenchymal-like phenotype which is distinct from the epithelioid phenotype of parental ELT3. Snail, a transcriptional repressor of E-cadherin, does not seem to respond to rapamycin in ELT3-245. Similarly, in ELT3-245 cells rapamycin does not seem to induce translocation of β-catenin to the cytoplasm, as opposed to the rapamycin-responsive ELT3 cells. Although β-catenin levels in the nucleus of untreated ELT3-245 cells are lower than those of untreated ELT3, it is possible that the consorted action of β-catenin and other transcription factors (e.g. Snail) confer a gene expression reprogramming capable to overcome response to rapamycin. Loss of E-cadherin and Occludin, increased expression of Snail, and resistance to anoikis has been previously reported in cells lacking Tsc2^48^. In light of the recent discovery of hamartin being a client and co-chaperon of Hsp90, the role of this interaction in rapamycin resistance would be worth exploring; increased Hsp90 expression has been linked to EMT in a variety of cancers^49–51^ Although Hsp90 is inhibited by rapamycin^52^, Hsp90 inhibitors synergize with rapamycin to induce cancer cell apoptosis^53^ possibly through activation of ER stress.

Additionally, we identified the ErbB signaling pathway to be up-regulated under rapamycin conditions, affecting both the MAPK and PI3K branch. MAPK signaling promotes estrogen-induced ELT3 lung metastasis, which is blocked by the ERK1/2 inhibitor CI-1040^34^. Finally, we found that the expression of integrins αV and β5 is elevated in ELT3-245 cells. High levels of integrins αV and β5 are associated with increased angiogenesis, vascular wall permeability, and migration^41–45^. Targeting molecules for αV and αVβ3 (e.g. intetumumab, abituzumab, and ProAgio^54–56^), which act as antiangiogenic agents, are in clinical trials for the treatment of metastatic melanoma, lung, and prostate cancer, primarily as adjuvants to cytotoxic agents.

The expression analyses for EMT-related and for Wnt/β-catenin and ErbB signaling genes signify an important aspect of ELT3-245; while expression of EMT-related genes (e.g. *Snai1*, *Ocln*, *Cldn*, *Jun*, *Myc*) is not affected by rapamycin treatment, genes for Wnt/β-catenin and ErbB signaling are differentially expressed in ELT3-245 only in the presence of rapamycin (e.g. *Ccnd1*, *Mapk3*, *Akt2*). Combined with the observation that rapamycin does not decrease ELT3-245 cell growth and colony size *in vitro* or tumor growth *in vivo*, it is likely that the rapamycin-resistant phenotypic changes are associated with increased expression of EMT-related genes, including oncogenic *Myc*, due to dysregulation of Wnt/β-catenin signaling. Nuclear β-catenin affects the expression of a variety of genes such as *Myc*, *Egfr*, *Cdh1*, *Snai1*, *Jun*, *Tjp1*, and *Ccnd1*^57–62^. Our data support such aberrant and rapamycin-insensitive β-catenin activation in the nucleus of ELT3-245 cells.

Consistent with the increased metastatic potential of ELT3-245, the levels and gelatinase activity of MMP2 and the expression of *Mmp2* were rapamycin-insensitive in these cells. Interestingly, untreated ELT3-245 had lower MMP2 activity, compared to ELT3, which can be partially explained by the decreased nuclear β-catenin in untreated ELT3-245 vs ELT3. Previous publications established a link between β-catenin signaling and MMP7, which consequently leads to the conversion of pro-MMP2 to active MMP2^63^. Second, β-catenin activates MT1-MMP (MMP14)^64^. Although neither *Mmp7* nor *Mmp14* were differentially regulated in ELT3-245 vs ELT3 cells, *Mmp15* (MMP15, MT2-MMP) was. Both MT1-MMP and MT2-MMP activate MMP2 and induce an invasive phenotype^65,66^. A third explanation would be the direct transcriptional regulation of *Mmp2* by β-catenin^67^.

The mesenchymal-like changes we observed in ELT3-245 cells are associated with the rapamycin-resistant phenotypes, but they are not necessarily causative in nature. Whether there are underlying genetic (in addition to the Tsc2 mutations) or epigenetic changes in ELT3-245 cells that differentiate them from ELT3 is unclear and certainly worthy of exploration. Several lines of evidence associate the TSC proteins with EMT and Wnt/β-catenin signaling. First, tuberin (TSC2) regulates E-cadherin localization to the plasma membrane via an AKT/mTORC1-dependent mechanism that is rapamycin-sensitive^48^. Our data of loss of E-cadherin and Claudin 1 and increased Snail in ELT3-245 are in agreement with this work. Second, hamartin and tuberin interacts with components of the β-catenin destruction complex, thus regulate Wnt/β-catenin signaling^68^. Therefore, it is possible that Wnt/β-catenin signaling in TSC-null cells is regulated by interactions between hamartin, tuberin, and the β-catenin destruction complex, and that perturbations in the stoichiometry or interactions of the component proteins can result in aberrant β-catenin degradation and persistent signaling to the nucleus.

Our analysis of size distribution for the rapamycin-treated ELT3 colonies, suggest that within ELT3 cultures there are two distinct cell populations; the majority of ELT3 cells are responsive to rapamycin, with a small percentage of cells that fail to respond to treatment. The heterogeneity of ELT3 cells in mediastinal metastases of placebo-treated mice and the observation that rapamycin treatment eliminates most, but not all, ELT3 cells from these tumors corroborate these *in vitro* findings. It is possible that the non-responsive ELT3 cells have enhanced mesenchymal-like characteristics, including loss of epithelial proteins with a concurrent gain of mesenchymal markers, and hyperactivation of the Wnt and MAPK signaling pathways. A previous study showed that LAM tissues have heterogeneous expression of Snail with low-expressing epithelioid-like cells, and high-expressing spindle cells^48^. This is in agreement with our hypothesis of a heterogeneous cell population for ELT3 cells. Alternatively, ELT3 cells may possess stem cell-like properties that, under rapamycin pressure, result in gene expression and signaling reprogramming. This reprogramming is capable of maintaining a rapamycin non-responsive (resistant) cell population within the TSC/LAM lesions. Stemness markers have been previously identified in TSC-related cell models and involve the interplay between Notch and Rheb^69^. Interestingly, we found that expression of *Dvl1* (Disheveled), an inhibitor of Notch signaling, was up-regulated in rapamycin-treated ELT3-245, compared to rapamycin-treated ELT3 cells.

In summary, our data support a new model for TSC and LAM pathogenesis and response to rapamycin treatment. This model postulates that a subset of TSC-null cells exhibits mesenchymal-like characteristics and/or stemness markers, and that these cells are either inherently resistant to rapamycin or become resistant due to gene expression and signaling reprogramming. Upon discontinuation of treatment and release of the mTORC1 blockade these “dormant” cells actively proliferate and rapidly re-establish the TSC/LAM tumors. These results may also have broader implications to cover other cancers with documented rapalog resistance (e.g. breast cancer, renal cell carcinoma, neuroendocrine tumors), and reinforce the role of aberrant Wnt/β-catenin signaling as a major facilitator for EMT-driven rapalog resistance.

## Methods

### Cell culture and treatments

ELT3 and ELT3-245 cells were cultured in IIA complete media (Supplementary Information section S.1.1). Rapamycin (Selleck Chemicals S1039) was dissolved in dimethyl sulfoxide (DMSO, Sigma D2650) and stored at −20 °C. Absolute counts of living and dead cells in cultures were obtained by the trypan blue exclusion methods on a Countess II FL Automated Cell Counter (Invitrogen).

### Protein analyses

Cell lysates were resolved in SDS-PAGE gels, transferred on PVDF membranes, and immunoblotted with primary and secondary antibodies. MMP2 activity was assayed by zymography. Detailed procedures are described in Supplementary Information section S.1.2.

### Xenograft studies

All mouse studies were approved by the Institutional Animal Care and Use Committees at the University of Tennessee Health Sciences Center (protocol #16-166), Texas Tech University Health Sciences Center (protocol #14031), and University of Cincinnati (protocol # TR01-15-07-22-01). All methods were carried out in accordance with the relevant guidelines and regulations. Eight-week-old female Fox Chase SCID (CB17 SCID) mice were obtained from Taconic (CB17SC-F EF) or The Jackson Laboratory (B6.CB17-Prkdcscid/SzJ). Where indicated, mice were ovariectomized by Taconic. Mice were inoculated subcutaneously with cells and when tumors grew they were treated three times a week with rapamycin by intraperitoneal injection of 3 mg/kg. Detailed procedures are described in Supplementary Information section S.1.3. Cell line ELT3-245 was established after removal of subcutaneous mouse tumor and tumor dissociation according to standard methods (see Supplementary Information section S.1.4). For short-term lung colonization studies^34^, mice and luciferase-expressing cells were pre-treated with rapamycin or vehicle control, inoculated intravenously with cells, and bioluminescence was measured at 1 h, 6 h and 24 h post inoculation (see Supplementary Information section S.1.5).

### Anchorage-independent cell growth (soft agar) assay

Each well of a 6-well tissue culture plate was coated with 1 ml 0.5% w/v agarose (Invitrogen) gel in 1x DMEM [DMEM (Corning MT10013CV), 10% v/v FBS, 100 U/ml penicillin and 100 μg/ml streptomycin] to form a bottom layer. For each well, 0.9 ml of cell suspension in 1x DMEM (2 × 10^4^ cells) was mixed with 1.05 ml of 1% w/v agarose and 1.05 ml of 2x DMEM (EMD Millipore SLM202B) at 37 °C, then directly added on top of the bottom layer. After the gel set, it was covered with 1 ml of 1x DMEM. Cultures were incubated at 37 °C in a humidified atmosphere containing 5% CO_2_. At the end of the culture period (2–3 weeks), the media were removed, and the agarose was overlaid with 1 ml 0.5% w/v iodonitrotetrazolium chloride (Sigma 58030) dissolved in 50% methanol. The cultures were incubated at 37 °C for 6 hours, then stored at 4 °C. Z-stack projection digital micrographs from 5 random fields were obtained in an EVOS FL Auto microscope (Invitrogen) using a 2x objective. Particle area (*A*) and circularity measurements for each colony were obtained with ImageJ (version 1.52 g) particle analysis tool, after applying auto-thresholding on Z-stack projections. Particle analysis parameters were: Size (pixels): 200-infinity; Circularity: 0.00–1.00. Colony radius (*r*) and volume (*V*) were calculated using the formulas [*r* = √ (*A*/π)] and [*V* = (4π*r*^3^)/3], respectively.

### Anchorage-independent cell death (anoikis) assay

Sub-confluent cells were trypsinized and re-suspended in media at a final concentration of 10^6^/ml. Four ml of cell suspension were plated in low-binding 6-well culture plates (Corning 3261) and harvested at the indicated timepoints. Cell death was measured by trypan blue exclusion. Cells were pelleted by centrifugation and lysed in PTY buffer for protein analysis.

### Gene expression studies

For microarray gene expression studies, triplicate samples were used for each cell line and treatment condition. Assays were performed on Clariom S rat-specific arrays (Affymetrix) using 1 μg total RNA. Fold-change (FC) for gene expression was calculated using standard methods. A FC threshold of 1.5 was applied to identify differentially expressed genes. The data were analyzed using Advaita Bio’s iPathwayGuide (https://www.advaitabio.com/ipathwayguide) to identify significantly impacted pathways. Pathway maps were obtained from the KEGG Pathway database^70^ (Kanehisa Laboratories). RT-qPCR was used to validate expression data of specific genes. Detailed methods are described in Supplementary Information section S.1.7.

### Image editing

Adobe Photoshop CC (Release 2017.0.1) was used for editing of digital images (immunoblots, gels, or micrographs). For presentation of final figures, digital images were cropped. Where indicated, intensity levels were modified throughout the entire cropped section. Unedited (raw) images are shown in Supplementary Information.

### Statistics and graphing

With the exception of gene expression array studies, all *in vitro* experiments were repeated at least three times. Statistical analysis and graphing were performed with Prism 7 (GraphPad).

## Supplementary information

Supplementary Information

Supplemental Data 1

Supplemental Data 2

Supplemental Data 3

## Data Availability

The gene expression data discussed in this publication have been deposited in NCBI’s Gene Expression Omnibus^71^ and are accessible through GEO Series accession number GSE119524 (https://www.ncbi.nlm.nih.gov/geo/query/acc.cgi?acc=GSE119524).
