# Supplementary material for: Rapalog resistance is associated with mesenchymal-type changes in Tsc2-null cells
Source: Sci Rep. 2019 Feb 28;9:3015. doi: 10.1038/s41598-019-39418-5 (PMC6395747; doi:10.1038/s41598-019-39418-5)

Rapalog resistance is associated with mesenchymal-type changes in Tsc2-null cells

Matthildi Valianou, Natalia Filippidou, Daniel L. Johnson, Peter Vogel, Erik Y. Zhang, Xiaolei Liu, Yiyang Lu, Jane J. Yu, John J. Bissler, Aristotelis Astrinidis

Supplementary Information

S.1. Supplementary Materials and Methods

S.1.1. Cell Culture Media. IIA complete media consist of DMEM/F12 50:50 (Sigma D6434 or Corning 16405CV), 50 nM Na_2SeO_3 (Sigma S9133), 1.6 µM $FeSO_4$ (Fisher Scientific I146), 25 µg/ml insulin (Invitrogen A11382IJ), 0.2 µM hydrocortisone (Sigma H4001), 10 µg/ml holotransferrin (Sigma T0665), 1 nM triiodothyronine (Sigma T2752), 10 µU/ml Arg8-vasopressin (Sigma V0377), 10 nM cholesterol (Sigma C3045), 20 ng/ml epidermal growth factor (Corning 354001)], 5 mM L-glutamine (Corning 25005CI), 10% v/v fetal bovine serum (FBS, GE Healthcare Life Sciences SH3007103 or Gibco A3160501), and 100 U/ml penicillin and 100 µg/ml streptomycin (Corning 30002CI).

S.1.2. Protein analyses. For immunoblotting of whole lysates, cells were washed once with cold phosphate-buffered saline (PBS, Invitrogen 21600044) and lysed in PTY buffer (50 mM HEPES, 50 mM NaCl, 5 mM EDTA, 1% Triton X-100, 50 mM NaF, 10 mM $Na_4P_2O_7$, 1 mM Na_3O_4V, 10 µg/ml phenylmethanesulfonyl fluoride) [1] supplemented with protease and phosphatase inhibitors (Sigma P8340, P0044, and P5726). For nuclear / cytoplasmic fractionation the Nuclear Extraction Kit (Sigma 2900) was used according to the manufacturer's instructions.

Total protein was quantified using BCA (Pierce 23227). 10-20 µg total protein per well were resolved in NuPage Bis-Tris gels (Invitrogen), transferred on Immobilon-P PVDF membrane (Millipore), and stained with Ponceau S solution (Sigma P7170) prior to blocking with 5% w/v bovine serum albumin (BSA, Sigma A7284) or 5% w/v non-fat dry milk (NFDM) dissolved in PBS containing 0.05% v/v Tween20 (Sigma P7949). Conditions for western immunoblotting are given in **Supplementary Table 1**. Secondary antibodies were HRP-conjugated mouse anti-rabbit or goat anti-mouse IgG (BioRad 1706516 and 1706515, respectively). Chemiluminescence was developed with SuperSignal West Pico (Thermo Fisher PI34078). Digital images of the chemiluminescence signal were captured on a Foto/Analyst FX 6-7206 (Fotodyne),

or on autoradiography film (USA Scientific 1968-3810), which was developed in an SRX-101A Film Processor (Konica) and then scanned.

For MMP2 activity, culture media were resolved in Novex Zymogram Plus gels (Invitrogen ZY00102BOX). After electrophoresis, gels were incubated in Novex Zymogram Renaturing Buffer (LC2670, Invitrogen) for 30 min at room temperature followed by a short incubation in Novex Zymogram Developing Buffer (LC2671, Invitrogen) for 30 min at room temperature and a longer incubation for 18 h at 37°C. Gels were washed 3 times with dH_2O for 5 min at room temperature and stained with SimplyBlue SafeStain (LC6060, Invitrogen) for 2 hours at room temperature. Finally, gels were destained in dH_2O for 2 h at room temperature, and images were acquired on a Foto/Analyst FX 6-7206.

For band intensity quantification, the ImageJ (version 1.52g) gel analysis tool was used on raw (unedited) images.

S.1.3. Xenograft studies. Mice were inoculated with 0.1 ml cell suspension of ELT3 or ELT3-245 cells in PBS ($2x10^7$ per ml) by subcutaneous injection in each of both flanks. After palpable tumors formed, tumor width (*W*) and length (*L*) were measured twice per week using digital calipers. Tumor volume (*V*) was calculated using the formula [$V = (W^2 \times L) / 2$] [2]. For treatments, rapamycin stock solution was prepared by dissolving 15 mg rapamycin (Selleck Chemicals S1039) in 1 ml DMSO and stored aliquoted at -20°C. Injectable rapamycin solution (0.6 mg/ml) was freshly prepared by dissolving 40 µl of the 15 mg/ml stock solution in 960 µl sterile normal saline (Becton Dickinson) containing 0.25% v/v Tween-80 (Sigma P8074) and 0.25% w/v PEG300 (Sigma 202371), or in 960 µl filter-sterilized corn oil (Sigma C8267). Mice were treated with rapamycin (3 mg/kg ip injection, 3 times per week) or vehicle control. Administration volume was 5 ml/kg body weight. At endpoint, mice were euthanized by CO_2 narcosis followed by thoracotomy and removal of the heart and lungs. Whole blood was collected by cardiac puncture of anesthetized animals into EDTA-Na_2 anticoagulant tubes. Primary tumors were divided in equal parts, which were stored in RNAlater Stabilizing Solution (Thermo Fisher AM7021), or fixed in 10% neutral buffered formalin (Fisher Scientific), or snap-frozen in liquid nitrogen then stored at -80°C. All other organs/tissues were fixed in 10% neutral buffered formalin and processed to paraffin embedding and sectioning [University of Tennessee Health Sciences Center (UTHSC) Research Histology Core].

S.1.4. Establishment of cell lines from xenograft tumors. The skin of euthanized animals was disinfected with 70% ethanol, and the tumors were aseptically removed. For tissue dissociation, all media were supplemented with 10 µg/ml ciprofloxacin HCl (Bioworld 403100311). A portion of the tumor was placed in a tissue culture plate in a small volume of

serum-free (SF) IIA complete and dissociated to < 1 mm^3 pieces by mechanical disruption with sterile scalpels. 5 ml of filter-sterilized 0.2% w/v collagenase type II (Worthington Biochemical Corporation LS004205) in SF IIA complete was added, and the tissue pieces were transferred into a 50 ml polypropylene tube. The tissue was washed three times with 0.2% w/v collagenase / SF IIA complete, resuspended in 5 ml 0.2% w/v collagenase / SF IIA complete and incubated at 37°C for 1-2 hours. Cells and tissue pieces were pelleted by centrifugation at 1,000 rpm for 10 min, and washed three times with 30 ml SF IIA complete without collagenase. The pellet was resuspended in IIA complete media supplemented with 5 mM L-glutamine, 15% v/v fetal bovine serum (FBS), 100 U/ml penicillin, 100 μg/ml streptomycin, and 10 μg/ml ciprofloxacin HCl, and cultured at 37°C in a humidified atmosphere containing 5% CO_2. Ciprofloxacin HCl was added to culture media during the first 10 passages of cells.

S.1.4.1 Determination of species of origin for isolated tumor cells. Genomic DNA from xenograft tumor-derived early-passage cultured cells was isolated using DNeasy Blood & Tissue Kit (Qiagen 69504). Rat- and mouse-specific primers and probes [3] were synthesized by Sigma and used in quantitative PCR (qPCR) amplification reactions with TaqMan™ Fast Advanced Master Mix (Thermo Fisher 4444557). Amplification, and data acquisition and analyses were carried out in an ABI 7500 Fast Real-Time PCR System (Applied Biosystems).

S.1.5. Short-term lung colonization studies. Luciferase-expressing ELT3-245 cells were generated after transfection with phCMV-CLUC (Genlantis P003500) and selection of individual clones in 1 mg/ml G418 (Invitrogen) for 2 weeks. Luciferase expression was assayed after cell lysis in Cell Culture Lysis Reagent (Promega E1531) using the Luciferase Assay System (Promega E1500) according to the manufacturer instructions. Relative luminescence was measured in a Synergy H1 multi-mode plate reader (Biotek). Luciferase-expressing ELT3 cells (ERL4) [3] were used as controls. Eight-week-old female CB17 SCID (The Jackson Laboratory, B6.CB17-Prkdcscid/SzJ) were pre-treated with a single dose of rapamycin (3 mg/kg ip), or vehicle control, for 48 hours. ERL4 and ELT3-245-luc-7 cells were pre-treated with 20 nM rapamycin or vehicle for 16 hours prior to intravenous inoculation in mice ($2x10^5$ cells in 0.1 ml PBS per mouse). At the time of inoculation, mice were treated with a second dose of rapamycin (or vehicle). Bioluminescence was measured after ip injection of XenoLight D-Luciferin (PerkinElmer 122799) using the Xenogen IVIS Spectrum In Vivo Imaging System (PerkinElmer) at 1 h, 6 h and 24 h post cell inoculation. Of note, we excluded animals that received poor luciferin administration during bioluminescence imaging.

S.1.6. Sequencing of *Mtor* FKBP12-rapamycin binding domain coding regions. NCBI Primer-BLAST was used to design intronic primers spanning each of exons 41-46 of rat *Mtor* (*Frap1*) using rat chromosome 5 genomic sequence as reference (accession number NC_005104.4) (**Supplementary Table 2**). Genomic DNA was extracted from exponentially growing ELT3 and ELT3-245 cells using DNeasy Blood & Tissue Kit (Qiagen 69504), and was quantified in a Synergy H1 plate reader using Take3 microvolume plate (Biotek). PCR was performed using Platinum Hot Start (Invitrogen 13000012) on a Veriti Fast 96-well thermal cycler (Applied Biosystems). Amplicons were analyzed on a 2% w/v agarose gel in 1x TAE buffer, and purified with Purelink PCR purification kit (Invitrogen K310001). Sanger sequencing was conducted by UTHSC Molecular Resource Center. Reactions were analyzed on an ABI Prism 3130XL Genetic Analyzer (Applied Biosystems). Sequences from both ELT3 and ELT3-245 were aligned to the corresponding sequences from the rat genome (sequence assembly Rnor_6.0) using Jalview 2.10.4b1.

S.1.7. Gene expression studies. Total RNA was isolated from exponentially growing cell cultures using RNeasy Mini kit (Qiagen 74104). RNA was quantified in a Synergy H1 multi-mode plate reader using a Take3 microvolume plate (Biotek). Samples with $OD_{260}/OD_{230} < 1.8$ were subjected to linear acrylamide / ethanol precipitation to increase purity.

Microarray gene expression assays were conducted by the UTHSC Molecular Resource Center. RNA integrity was assessed on a 2100 Bioanalyzer System using an RNA 6000 Nano Kit (Agilent). Assays were performed from 1 μg total RNA on Clariom S rat-specific arrays (Affymetrix). Arrays were scanned on a GeneChip Scanner (Affymetrix). Microarray gene expression data analysis was conducted by the UTHSC Molecular Bioinformatics Core. Fold-change (FC) for gene expression was calculated using standard methods. A FC threshold of 1.5 was applied to identify differentially expressed genes. A Welch *t* test and Benjamini – Hochberg false discover rate were used to calculate statistical significance. Genes with an adjusted p-value ≤ 0.05 where considered statistically significant. Pathway analyses were performed using iPathwayGuide (Advaita).

For gene expression studies by RT-qPCR, the SuperScript VILO cDNA Synthesis Kit (Invitrogen 11754050) was used to synthesize cDNA from 1 μg total RNA in a reaction volume of 20 μl. The cDNA synthesis reaction was diluted 1:4 with nuclease-free H_2O (Ambion AM9930), and 2 μl of diluted cDNA was used for target amplification using the TaqMan Fast Advanced Master Mix (Applied Biosystems 4444557) and gene-specific TaqMan assays (**Supplementary Table 3**) in technical triplicates. *Actb* (β-actin) was used as housekeeping gene. Amplification and detection were

performed in an ABI 7500 Fast Real-Time PCR System. Pairwise fold-change (FC) differences were calculated using the $\Delta\Delta$Ct (Livak) method [4,5]. FC values less than 0.5 or greater than 1.5 were considered significant.

S.2. Supplementary Tables

Supplementary Table 1. Primary antibodies used for immunoblotting.

Protein/epitope	Species	Supplier	Cat #	Blocking	1º ab dilution
β-Actin	Mouse	Sigma	A5441	5% NFDM [1]	1:10,000
pS473-AKT	Rabbit	CST [2]	9271	5% BSA [3]	1:1,000
Pan-AKT	Rabbit	CST	4691	5% NFDM	1:1,000
Bim	Rabbit	CST	2933	5% NFDM	1:1,000
E-Cadherin	Mouse	CST	14472	5% NFDM	1:1,000
β-Catenin	Rabbit	CST	8480	5% NFDM	1:1,000
Claudin	Rabbit	CST	13255	5% NFDM	1:1,000
Cleaved Caspase 3	Rabbit	CST	9664	5% NFDM	1:750
GAPDH	Mouse	Millipore	MAB374	5% NFDM	1:1,000
MMP2	Rabbit	Abcam	ab92536	5% NFDM	1:1,000
pS2448- mTOR	Rabbit	CST	5536	5% BSA	1:1,000
PCNA	Rabbit	CST	13110	5% BSA	1:500
pS235/236-S6	Rabbit	CST	2212	5% BSA	1:10,000
Total S6	Mouse	CST	2317	5% NFDM	1:1,000
pT389-p70S6K	Rabbit	CST	9234	5% BSA	1:1,000
Snail	Rabbit	CST	3879	5% NFDM	1:500
α-Tubulin	Mouse	Sigma	T9026	5% NFDM	1:100,000
ZO-1	Rabbit	CST	8193	5% NFDM	1:1,000

[1] NFDM: non-fat dry milk

[2] CST: Cell Signaling Technology

[3] BSA: bovine serum albumin (Sigma A7284)

Supplementary Table 2. Primer information for amplification of rat *Frap1* (mTOR) exons 41-46.

Exon	Forward primer sequence (5' -> 3')	Reverse primer sequence (5' -> 3')	Amplicon length (bp)
41	ATCTTTCCTATGTCTCATGCTGC	AAATGCTTAGGGTCTGCACG	169
42	CATCGCTTTGGTGGCCTTTT	CACCCTCCTGGCTGCTG	195
43	TTCCTGTGGTGTTTCCTTCCTC	AGGGTACTGTCCTGTCACTCTC	253
44	GCGATAACTTGAAACTTTGTTCTGC	GACAGACAACCCTGTTGGGA	206
45	AGAGGCAGAAGTCTAAGTCCTTT	GCCGTCACTTAAGAAGAGATGA	253
46	GTGTGGCTCTGATCTATTTCTGT	GAGCTCAGCTAGGACACACG	208

Supplementary Table 3. TaqMan assays of gene expression analysis of target genes by RT-qPCR.

Gene Symbol	Protein	Assay #
Actb	β-actin	Rn00667869_m1
Axin2	Axin 2	Rn00577441_m1
Cldn1	Claudin 1	Rn00581740_m1
Egfr	Epidermal growth factor receptor	Rn06299372_s1
Jun	c-Jun	Rn00572991_s1
Mmp2	Matrix metalloproteinase 2	Rn01538170_m1
Myc	c-Myc	Rn00561507_m1
Ocln	Occludin	Rn00580064_m1
Snai1	Snail	Rn00441533_g1
Snai2	Slug	Rn01404476_m1

S.3. Supplementary References

1 O'Neill, G. M. & Golemis, E. A. Proteolysis of the docking protein HEF1 and implications for focal adhesion dynamics. *Mol Cell Biol* **21**, 5094-5108, doi:10.1128/MCB.21.15.5094-5108.2001 (2001).

2 Faustino-Rocha, A. *et al.* Estimation of rat mammary tumor volume using caliper and ultrasonography measurements. *Lab Anim (NY)* **42**, 217-224, doi:10.1038/laban.254 (2013).

3 Yu, J. J. *et al.* Estrogen promotes the survival and pulmonary metastasis of tuberin-null cells. *Proc Natl Acad Sci U S A* **106**, 2635-2640, doi:10.1073/pnas.0810790106 (2009).

4 Livak, K. J. & Schmittgen, T. D. Analysis of relative gene expression data using real-time quantitative PCR and the 2(-Delta Delta C(T)) Method. *Methods* **25**, 402-408, doi:10.1006/meth.2001.1262 (2001).

5 Schmittgen, T. D. & Livak, K. J. Analyzing real-time PCR data by the comparative C(T) method. *Nat Protoc* **3**, 1101-1108 (2008).

S.4. Supplementary Figures

Figure S1. Rapamycin has no significant effect on ELT3-245 growth *in vitro*. (A) ELT3 and ELT3-245 cells were plated in 35 mm tissue culture plates (10^4 cells / plate, n = 3), treated with DMSO or 100 nM rapamycin for four days and the number of living cells was counted. **(B)** Growth of ELT3 and ELT3-245 cells at four days in the absence and presence of 100 mM rapamycin. Y-axis values are normalized growth to the DMSO-treated cells within each group.

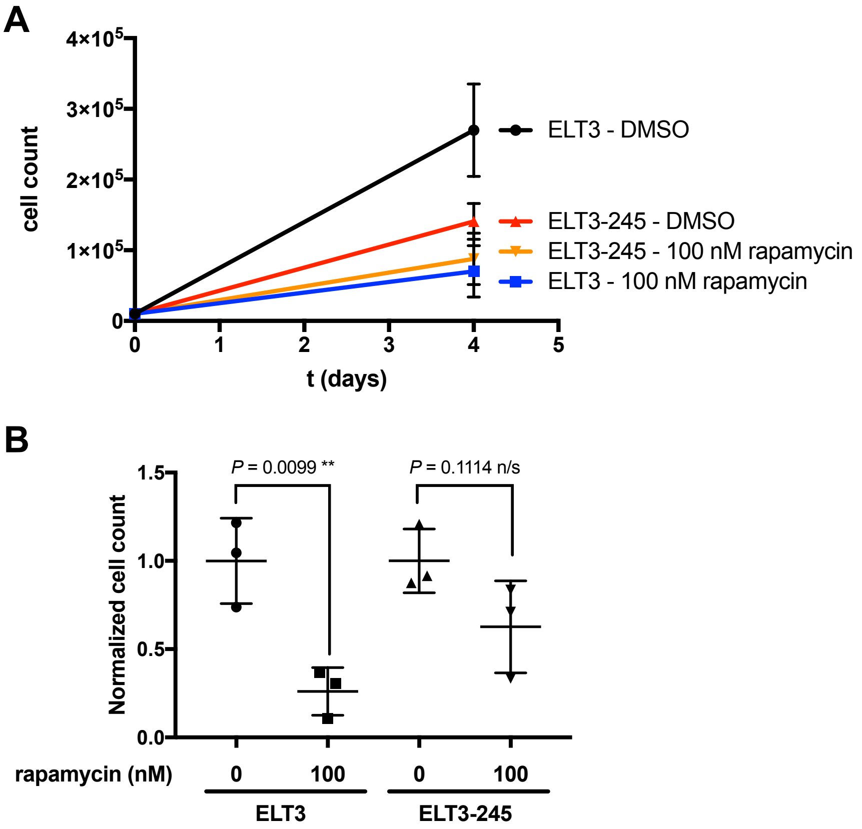

Figure S2. Rapamycin effect on mTORC1 and mTORC2 signaling in ELT3 and ELT3-245 cells. Sub-confluent ELT3 and ELT3-245 cells were treated with vehicle control (0 nM rapamycin), 0.2 nM rapamycin, or 2 nM rapamycin for 24 hours. Lysates were analyzed by PAGE and immunoblotting for the indicated antibodies. 0.2 nM rapamycin caused a partial dephosphorylation of ribosomal protein S6 in ELT3 cells and to a lesser extent in ELT3-245 cells. 2 nM rapamycin caused a complete S6 dephosphorylation in ELT3 cells; however, residual phosphorylation was observed in lysates from ELT3-245 cells treated with 2 nM rapamycin. Cropped sections of blots are shown.

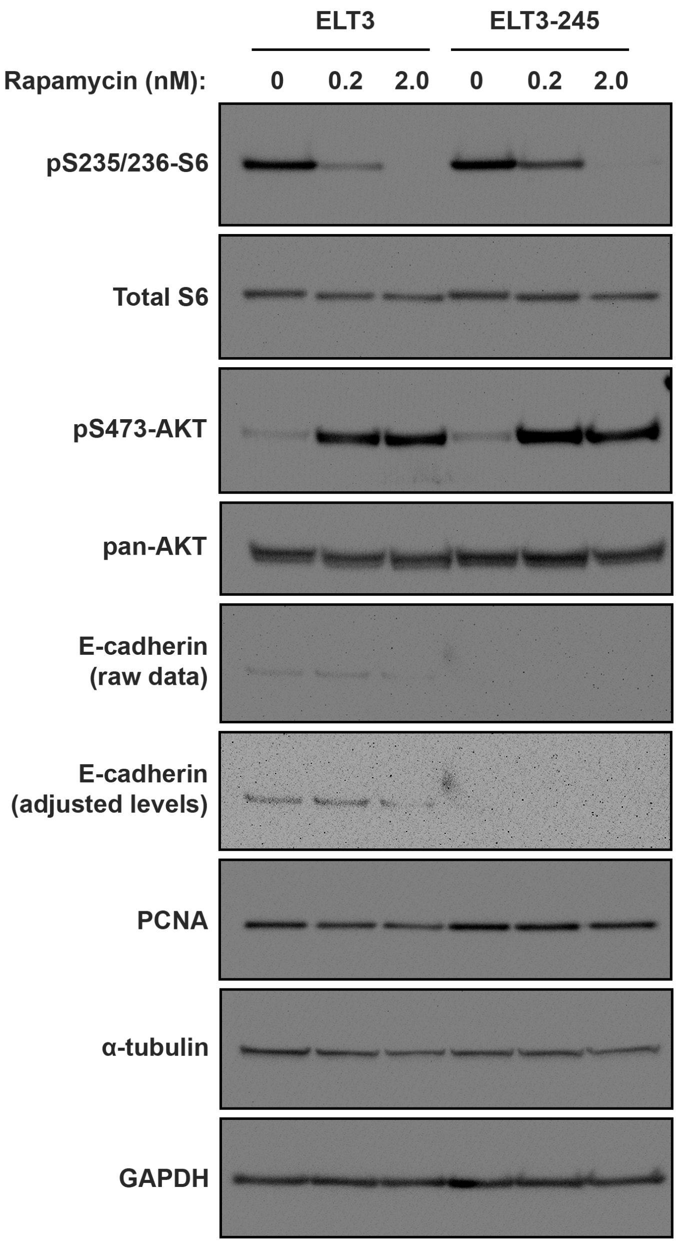

Figure S2. (continued). Raw (unedited) digital images of chemiluminescence (left) and brightfield (right). Black dashed-line rectangles correspond to the cropped immunoblot sections.

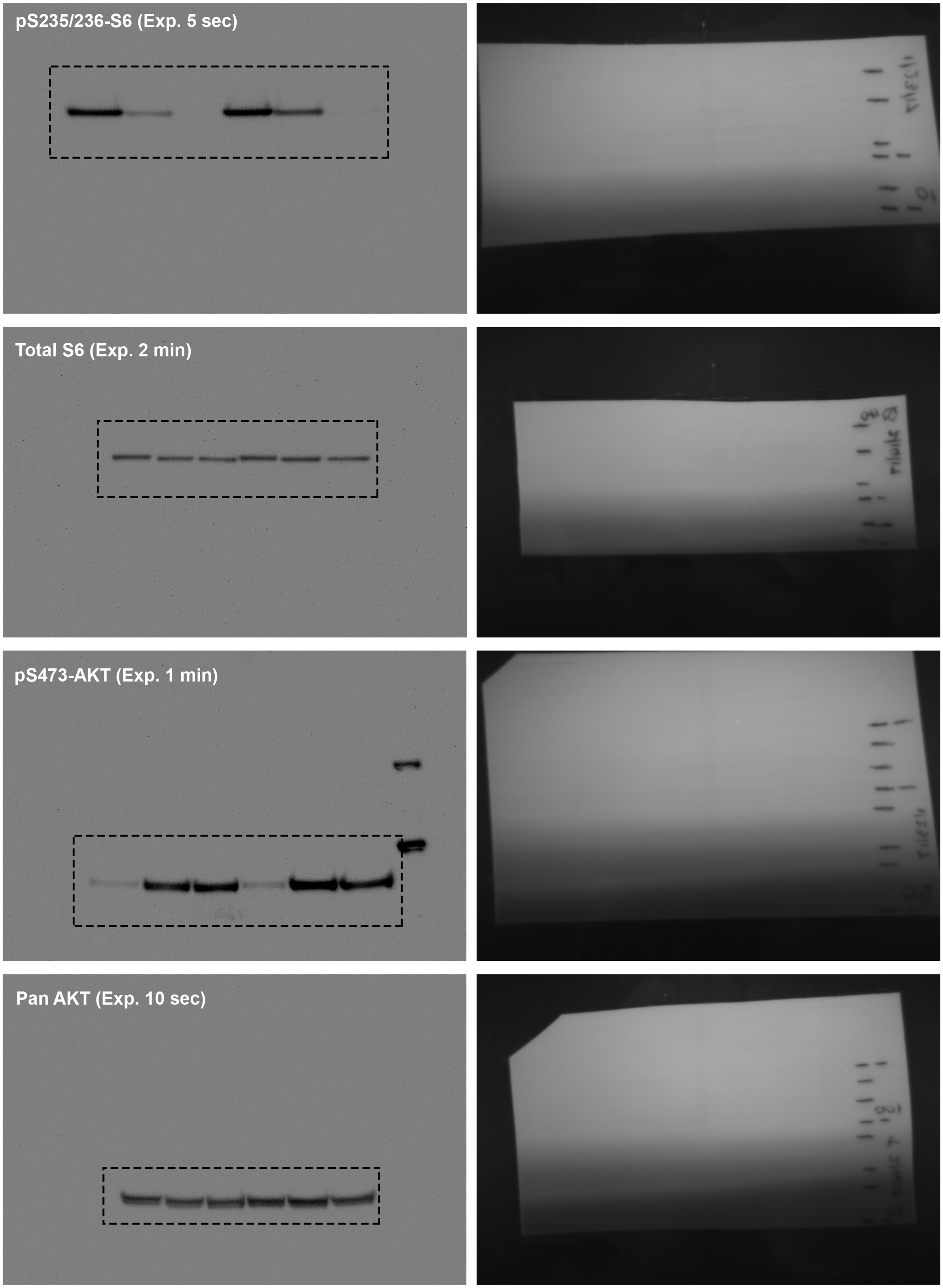

Figure S2. (continued). Raw (unedited) digital images of chemiluminescence (left) and brightfield (right). Black dashed-line rectangles correspond to the cropped immunoblot sections.

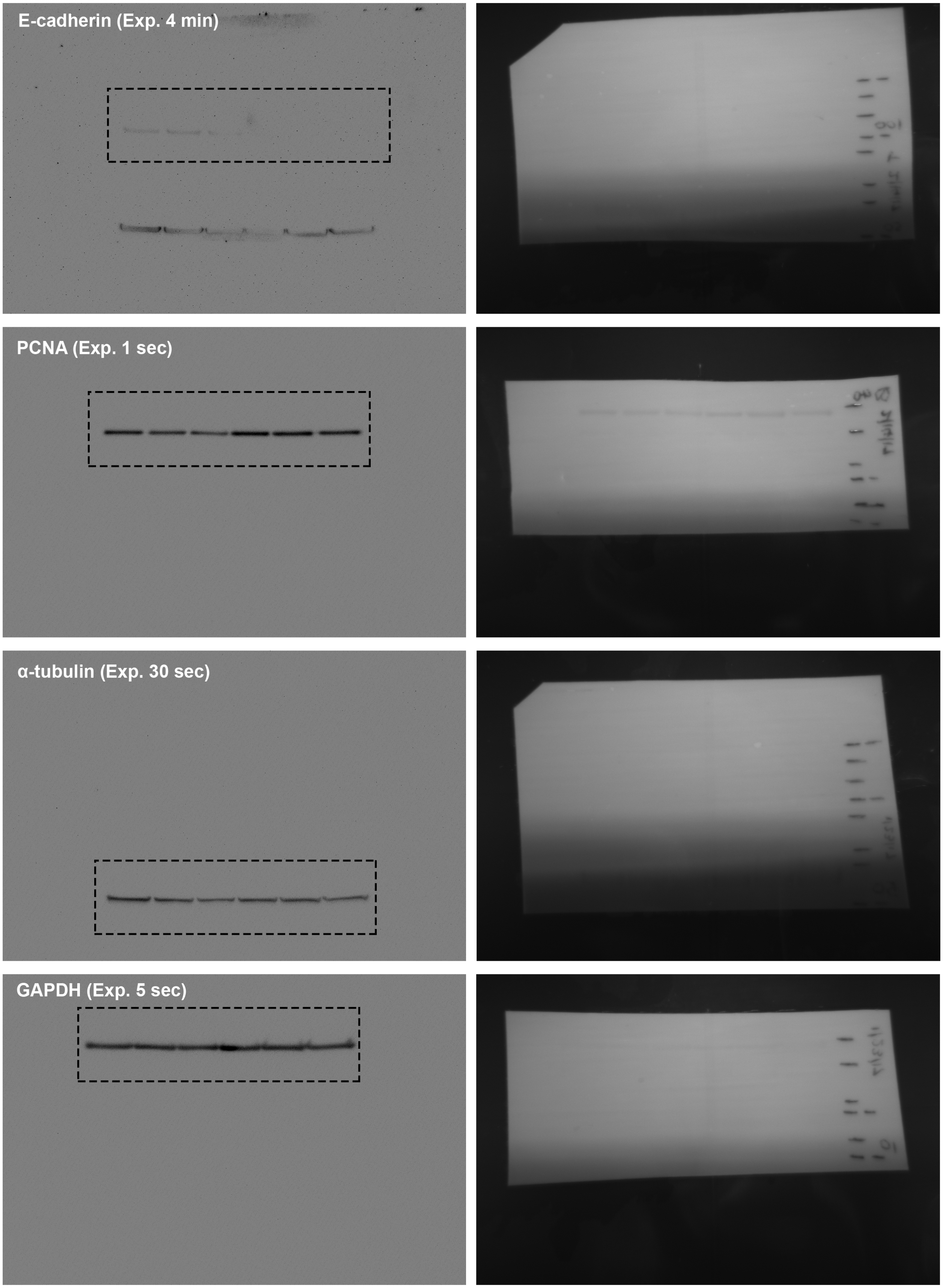

Figure S3. Supplementary images for Fig. 1D. Digital images of chemiluminescence (left) and brightfield (right) were obtained with a Foto/Analyst FX 6-7206 (Fotodyne). Raw (unedited) images are shown. Black dashed-line rectangles correspond to the cropped immunoblot sections shown in Fig. 1D. White dashed lines indicate the membrane boundaries.

pS235/236-S6 (Exp. 5 sec)

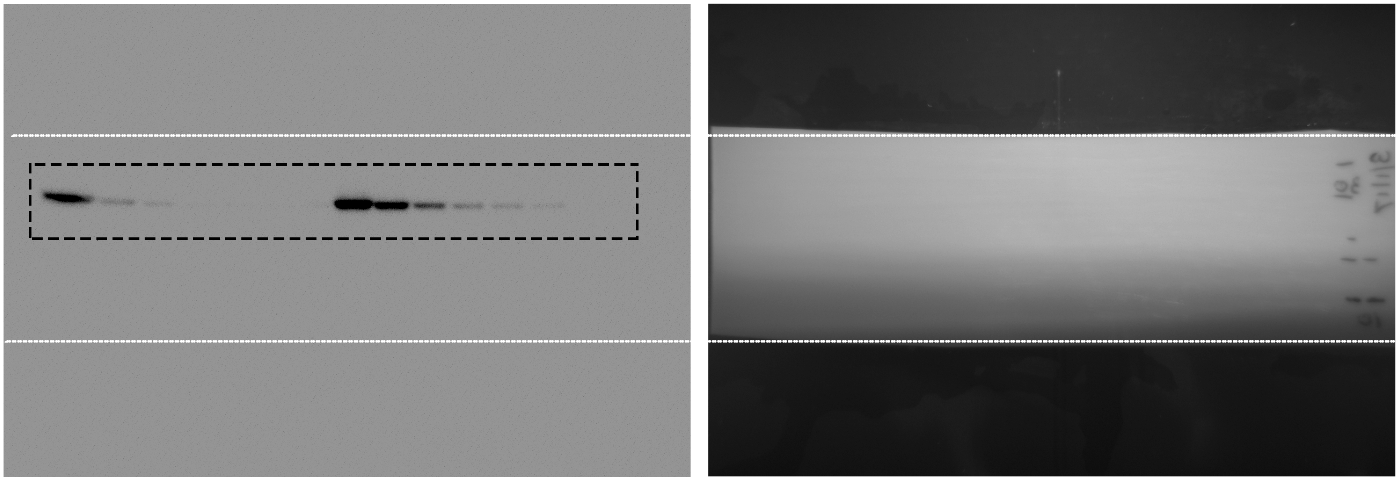

Total S6 (Exp. 30 sec)

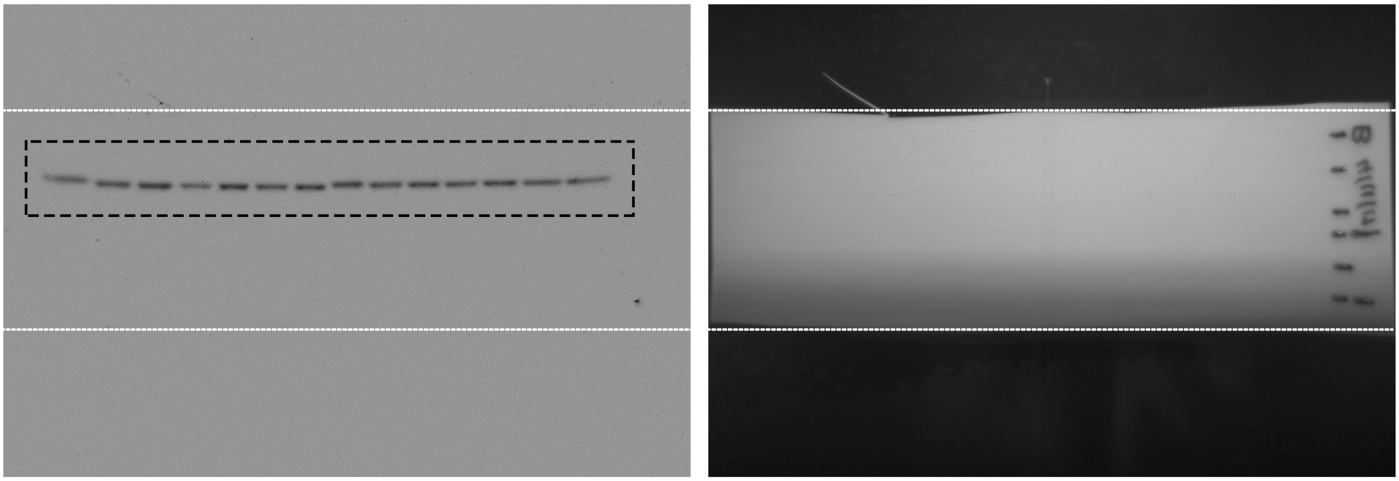

GAPDH (Exp. 1 sec)

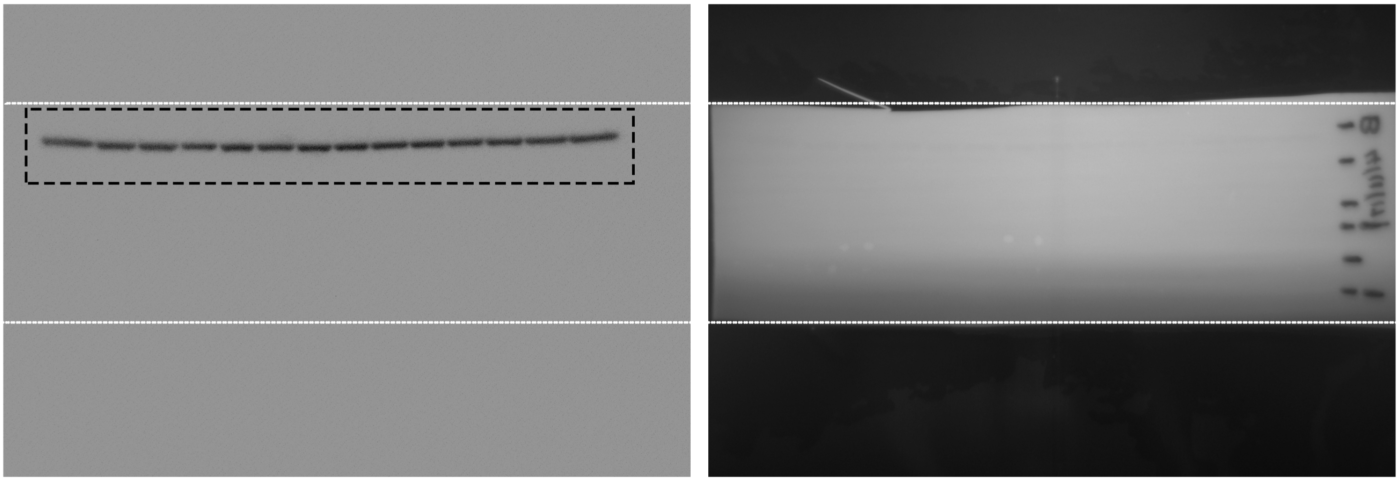

Figure S3. Supplementary images for Fig. 1D. (continued)

Pan AKT (Exp. 1 sec)

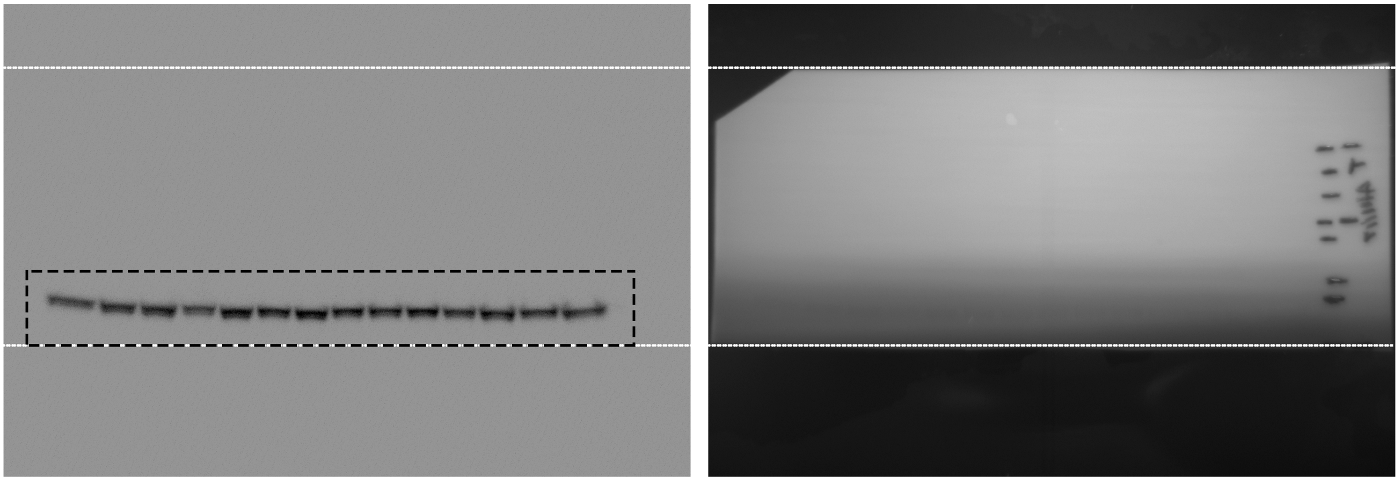

pS473-AKT (Exp. 2 min)

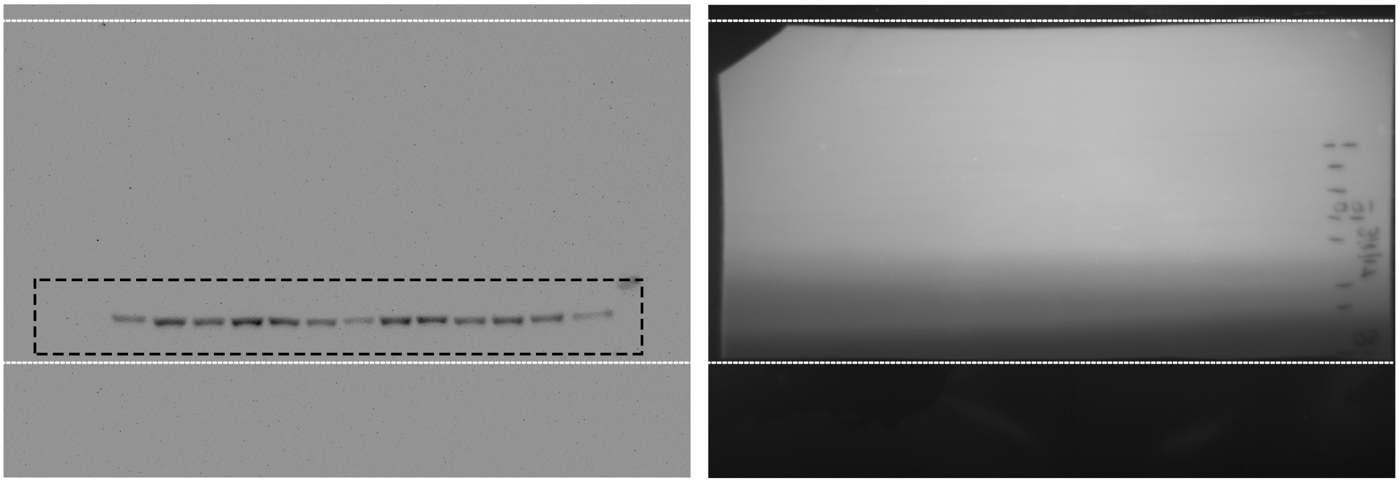

α-tubulin (Exp. 30 sec)

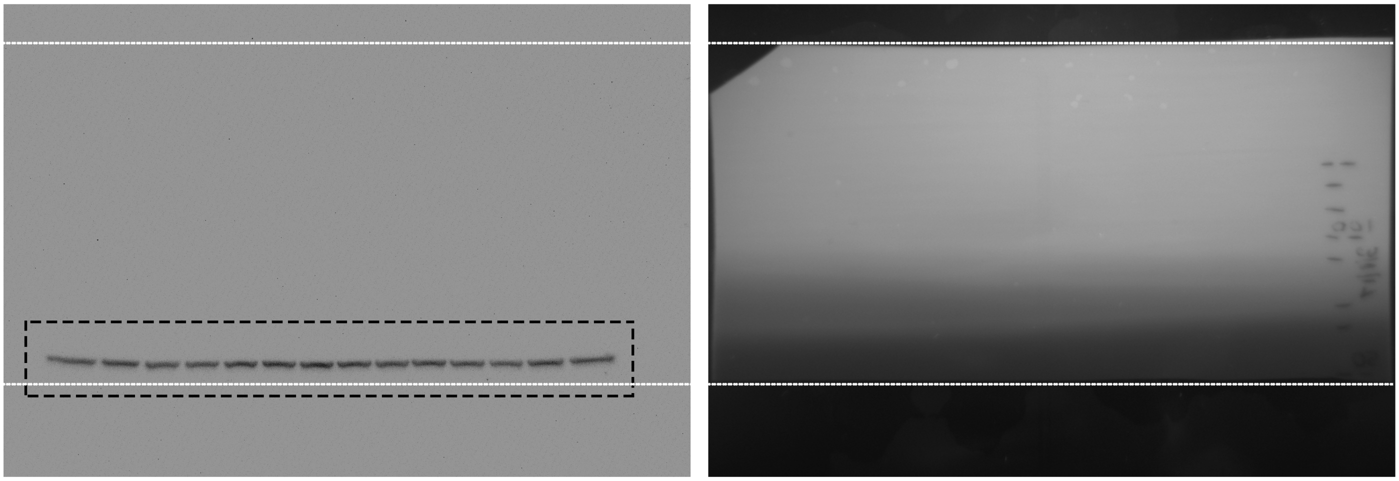

Figure S4. ELT3-245 tumors do no respond to rapamycin treatment *in vivo*. (A) ELT3-245 tumor-bearing mice were treated with rapamycin (3 mg/kg ip, 3 times per week). Tumors partially, but not significantly responded to rapamycin between days 1 and 8 (day 1: 367.5 mm^3 ± 40.64, n = 15; day 8: 303.1 mm^3 ± 50.33, n = 15; P = 0.3277). ELT3-245 tumor volume significantly increased by day 22, compared to day 1 (day 1: 367.5 mm^3 ± 40.64, n = 15; day 22: 856.7 mm^3 ± 177.7, n = 10; $P = 0.0038 < 0.01$). **(B)** Tumor volume scatter plots of ELT3 and ELT3-245 tumor-bearing mice at days 1, 8 and 22, in the two treatment cohorts (rapamycin, placebo). ELT3-245 tumors were significantly bigger at day 22 in the rapamycin treatment cohort, compared to ELT3 cells (ELT3: 187.4 mm^3 ± 138.1, n = 4; ELT3-245: 856.7 mm^3 ± 177.7, n = 10; $P = 0.0451 < 0.05$).

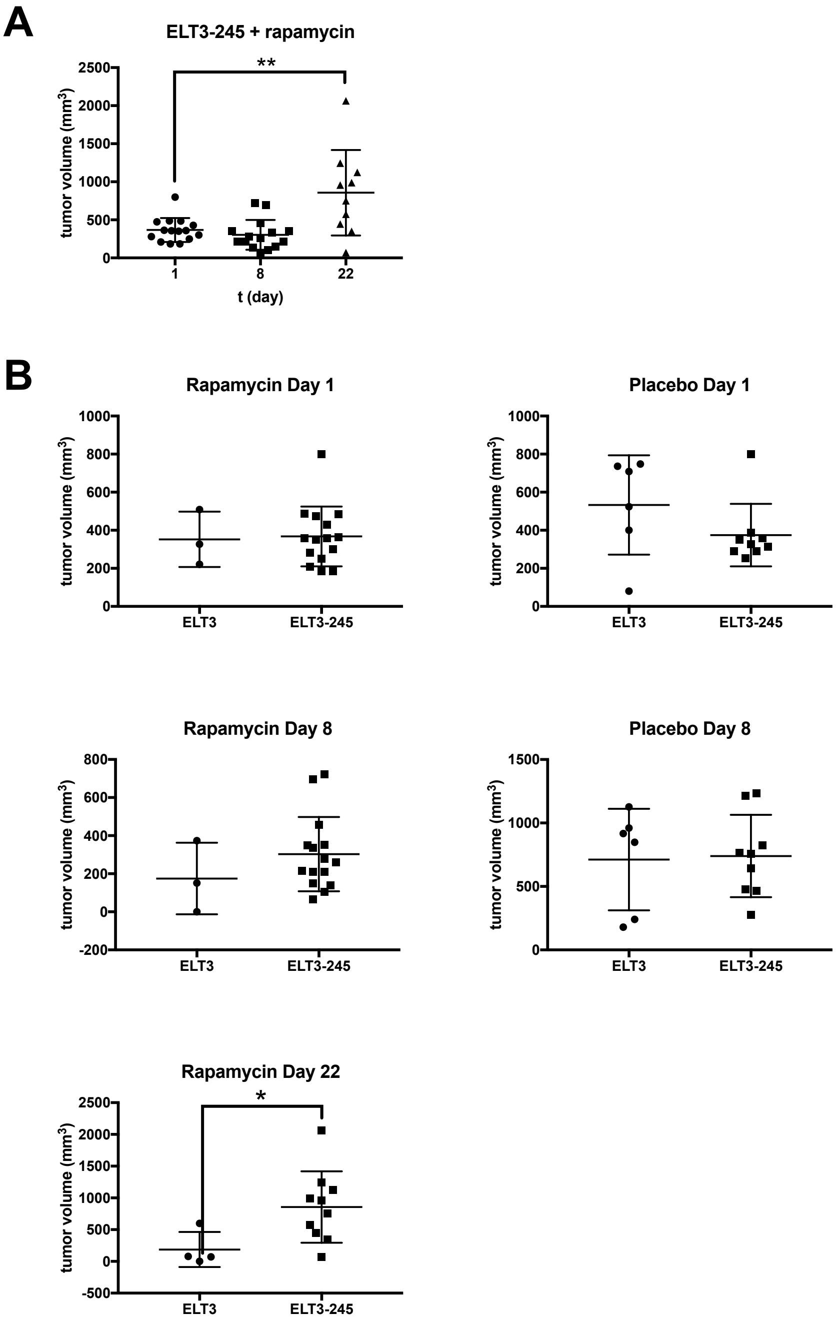

Figure S5. **ELT3-245 exhibit enhanced anchorage-independent cell growth.** ELT3 and ELT3-245 cells were embedded in 0.3% w/v agarose in triplicate wells of a 6-well plate (10^4 cells per well) and allowed to form colonies for 13 days. Phase-contrast micrographs were obtained with a 4x objective. **(A)** Representative phase-contrast micrographs of ELT3 and ELT3-245 colonies. Scale is 125 μm. **(B)** Particle analysis of colonies at day 13 post inoculation. ELT3-245 cells formed significantly bigger colonies (4,356,563 μm^3 ± 862,070, n = 21), compared to ELT3 cells (362,423 μm^3 ± 89,236.7, n = 18), $P < 0.001$.

A

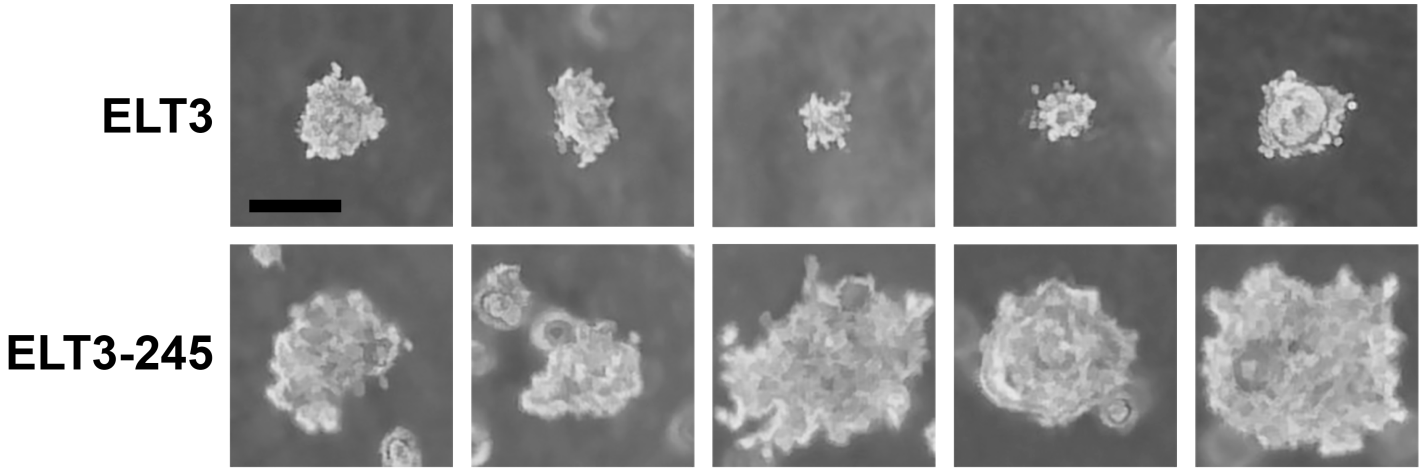

B

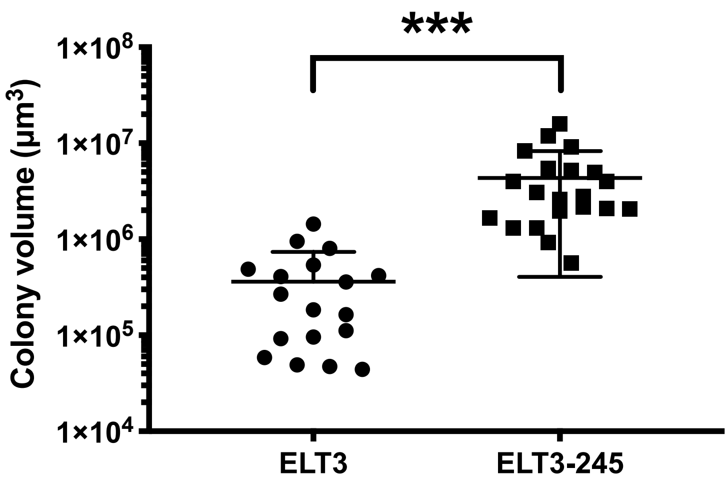

Figure S6. Supplementary images for Fig. 2F. Digital (scanned) images of chemiluminescence signal captured on autoradiography film. Raw (unedited) images are shown. Black dashed-line rectangles correspond to the cropped immunoblot sections shown in Fig. 2F.

Bim (Exp. 10 sec)

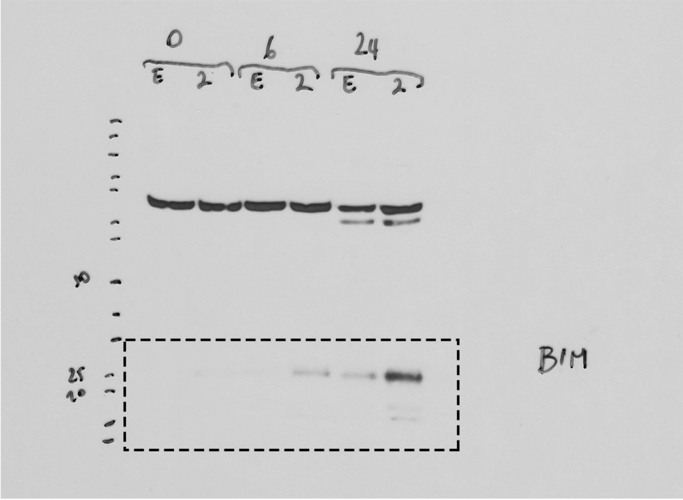

Cleaved Caspase 3 (Exp. 2 min)

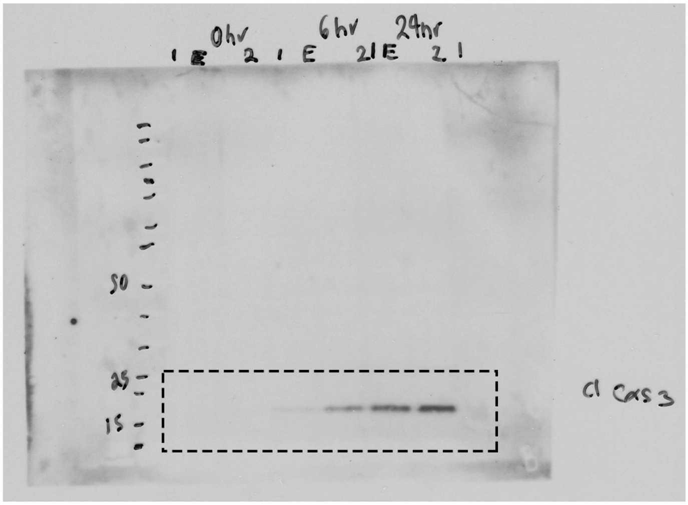

Bim (Exp. 40 sec)

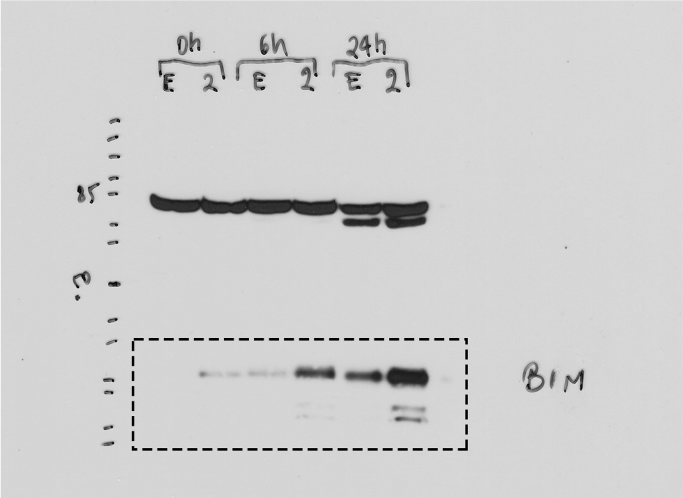

α-tubulin (Exp. 15 sec)

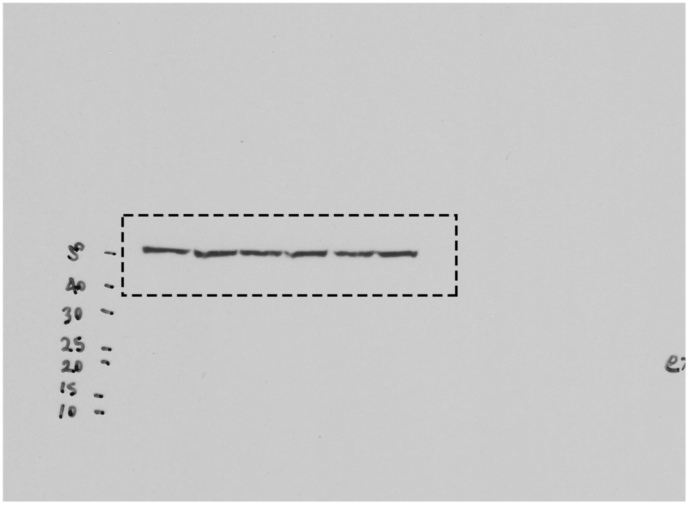

Figure S7. Rapamycin does not decrease lung tumor burden in ELT3-245 tumor-bearing mice. Micrographs (40x objective) of hematoxylin-eosin stained sections from lung mediastina of ELT3 (a, b) and ELT3-245 (c, d) tumor-bearing mice treated with vehicle (a, c) or rapamycin (b, d). a. Arrows indicate large anaplastic cells, arrowheads indicate the position of smaller cells with lower cytoplasmic content. b. Arrows indicate apoptotic cells.

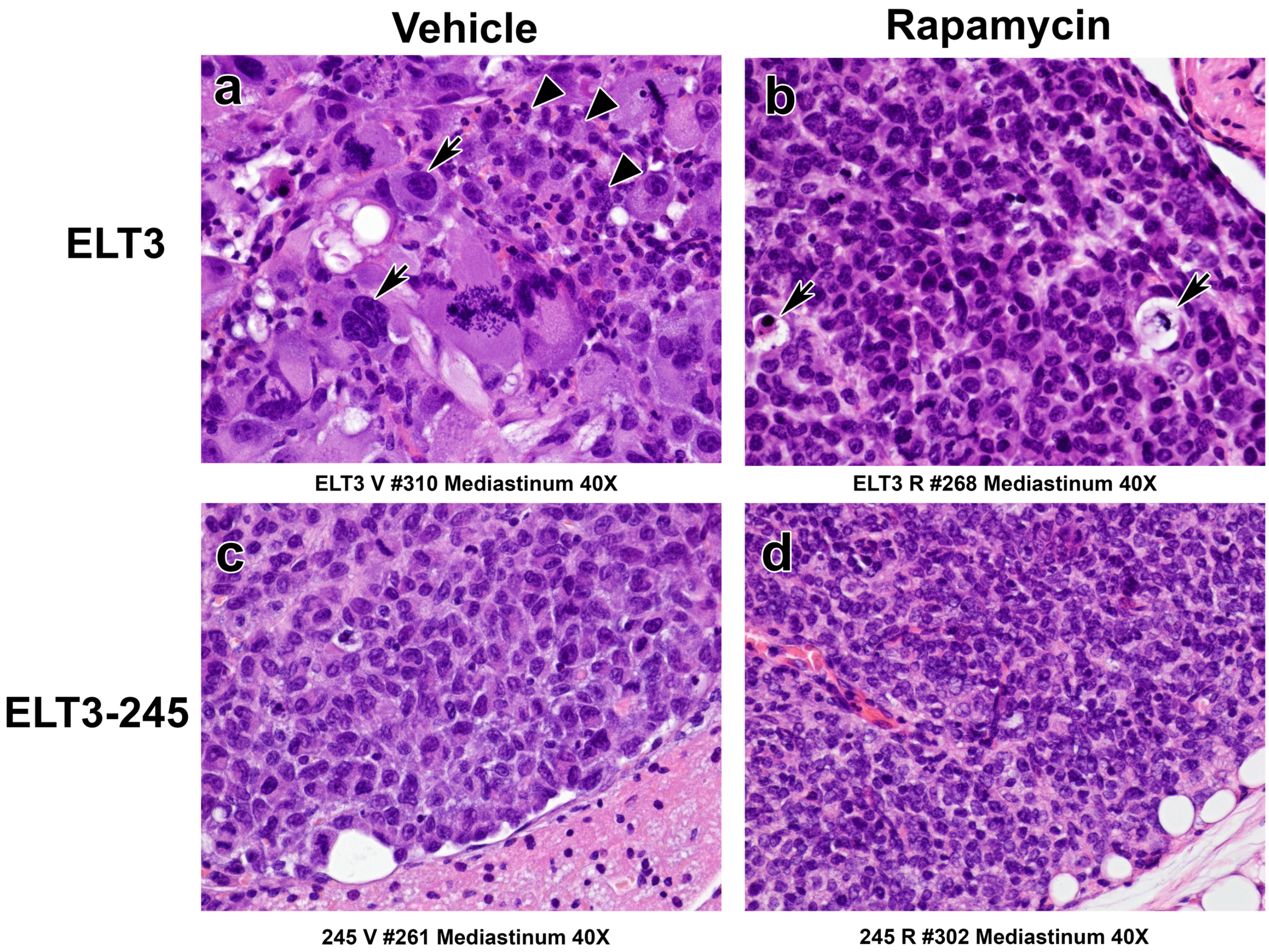

Figure S8. Rapamycin decreases short-term lung colonization by ELT3-245 cells. SCID mice were treated with vehicle or rapamycin (3 mg/kg ip) 48 hours prior to cell inoculation. ELT3-245-luc-7 cells were pre-treated with vehicle or 20 nM rapamycin for 16 hours, then inoculated by tail-vein injection in mice. At time of inoculation, a second dose of vehicle or rapamycin was administered to the mice. Photon flux was normalized to baseline for each mouse. Normalized photon flux at 24 h post cell inoculation: Vehicle: 1.388 ± 0.09044, $n = 3$; Rapamycin: 0.6496 ± 0.1341, $n = 3$; $P = 0.0103$.

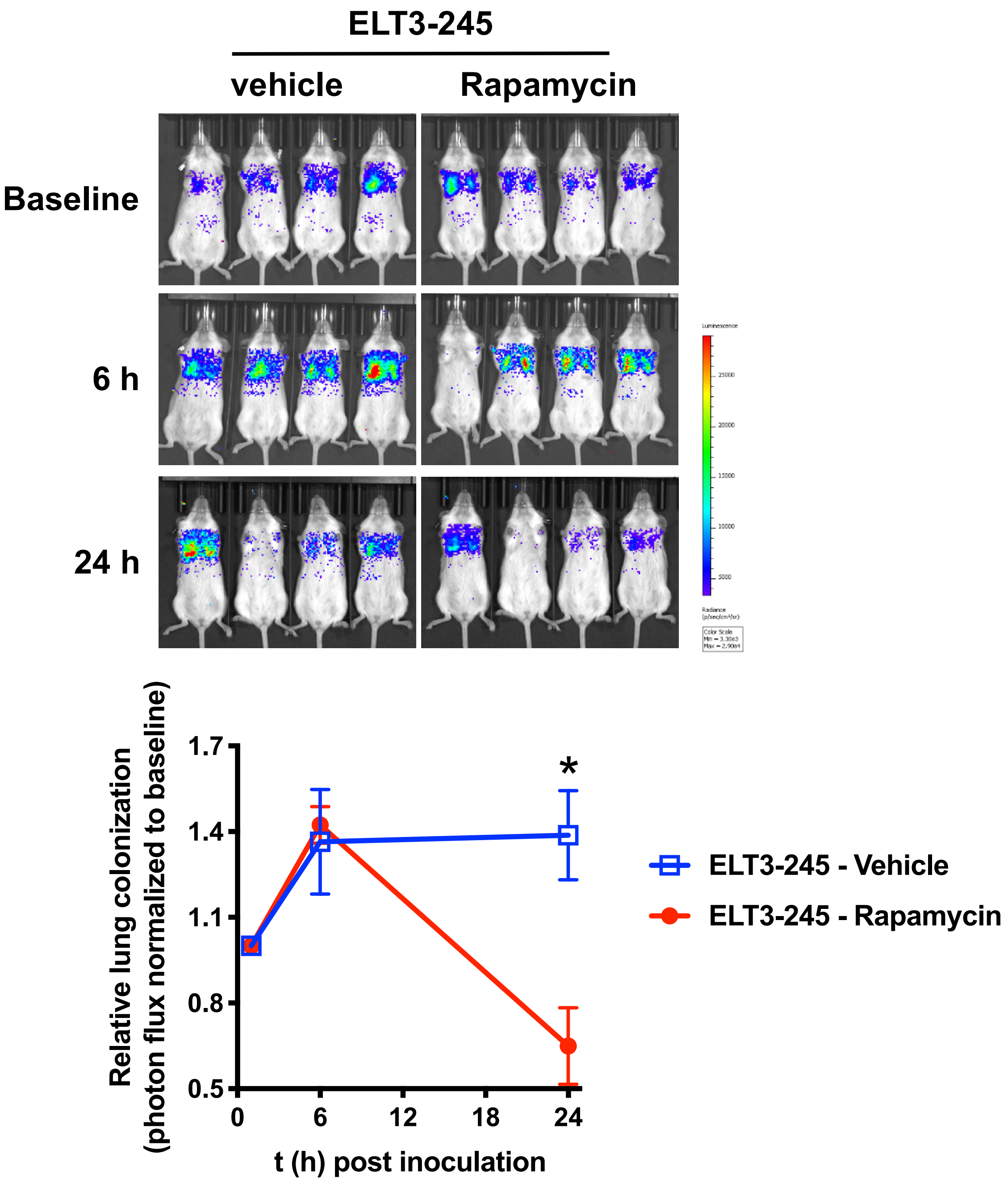

Figure S9. Supplementary images for Fig. 4A. Digital (scanned) images of chemiluminescence signal captured on autoradiography film. Raw (unedited) images are shown. Black dashed-line rectangles correspond to the cropped immunoblot sections shown in Fig. 3A. White dashed lines indicate the membrane boundaries. This page: blots for pS235/236-S6, ZO-1, β-catenin, Claudin. Next page: blots for Snail, E-cadherin, GAPDH.

Gel 1 (Exp. 1 sec)

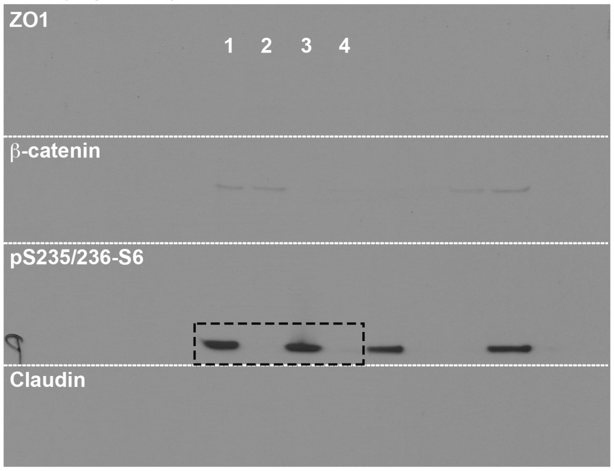

Gel 1 (Exp. 40 sec)

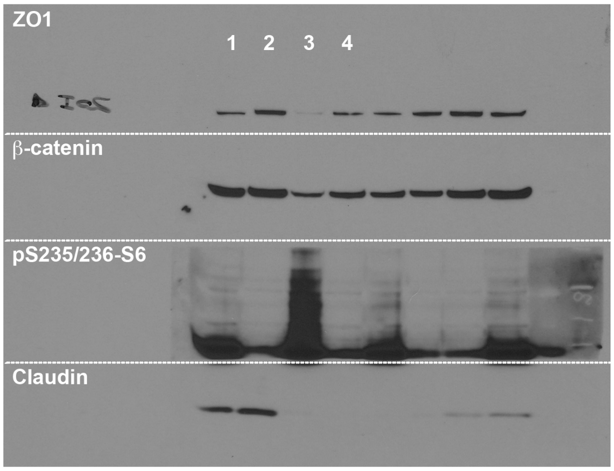

Gel 1 (Exp. 10 sec)

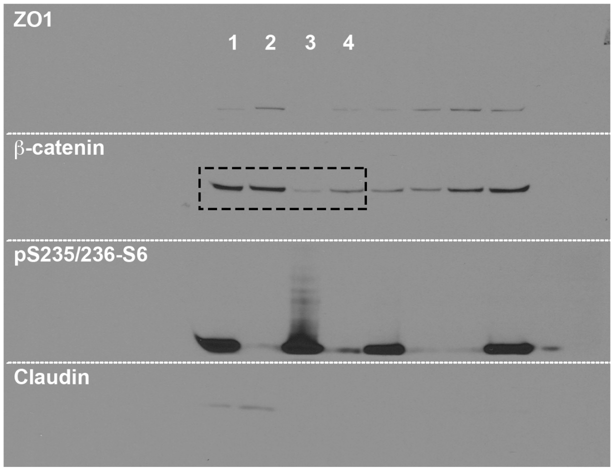

Gel 1 (Exp. 2 min)

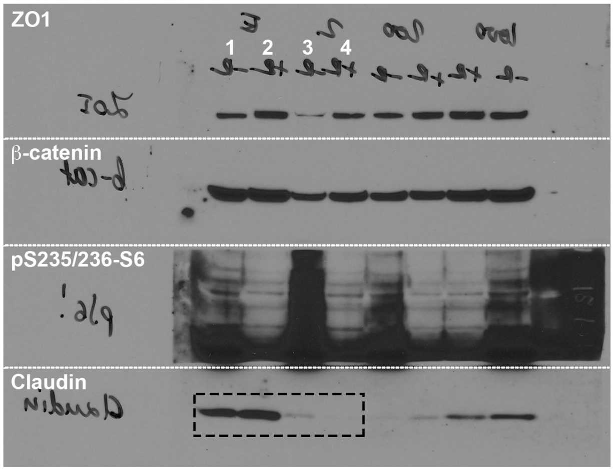

Gel 1 (Exp. 15 sec)

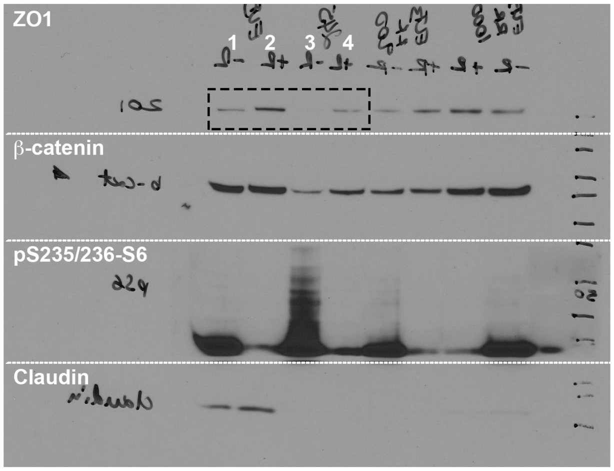

Gel 1 re-probe (Exp. 8 sec)

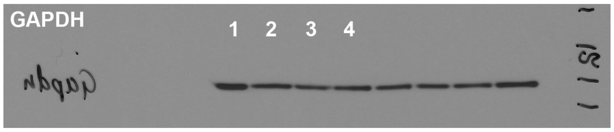

Figure S9. Supplementary images for Fig. 4A. (continued) The rabbit E-cadherin antibody (CST #3195) is not cross-reactive for rat E-cadherin. The mouse E-cadherin antibody (CST #14472) is cross-reactive for rat E-cadherin.

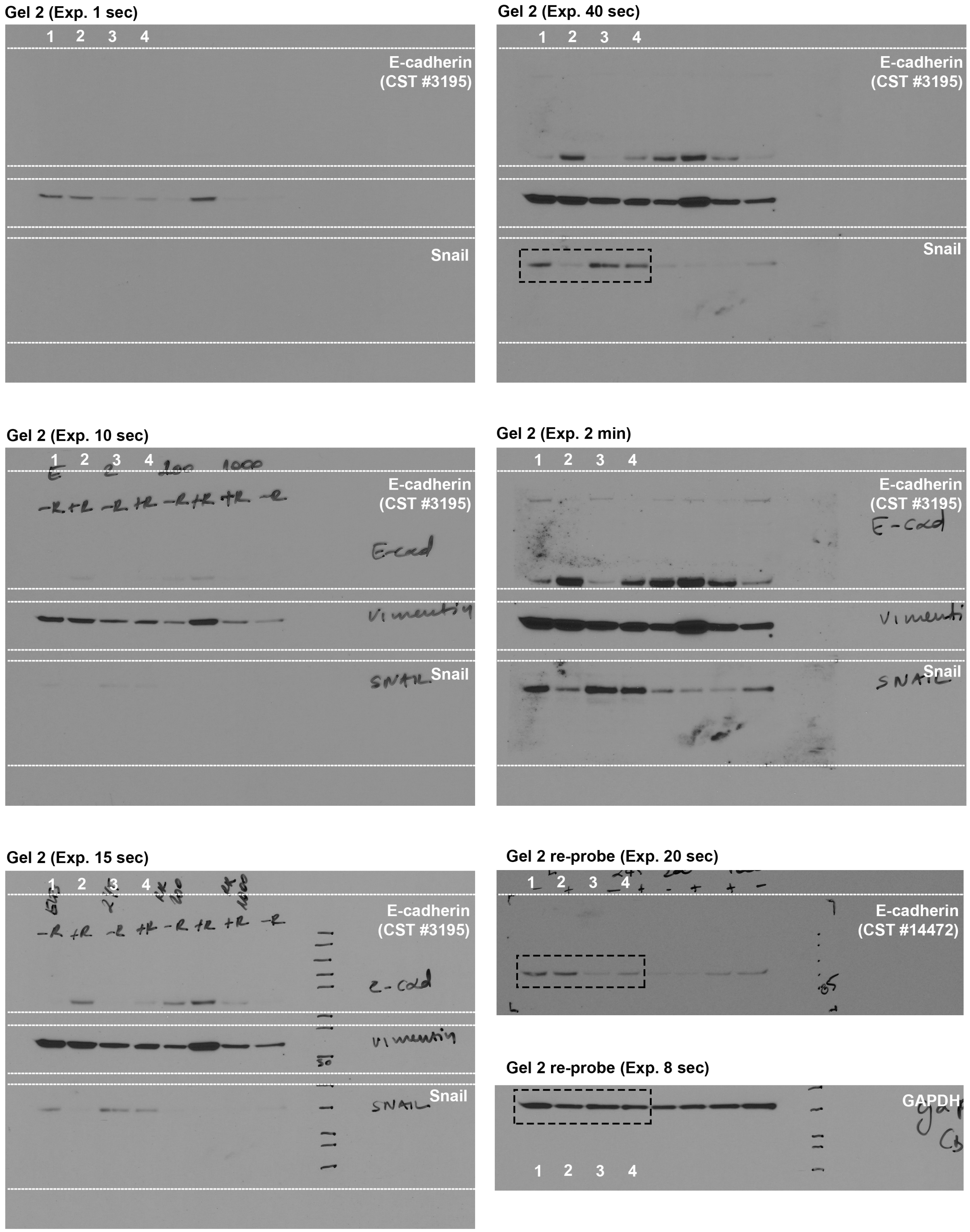

Figure S10. Supplementary images for Fig. 4C. Digital (scanned) images of chemiluminescence signal captured on autoradiography film. Raw (unedited) images are shown. Black dashed-line rectangles correspond to the cropped immunoblot sections shown in Fig. 3C. White dashed lines indicate the membrane boundaries.

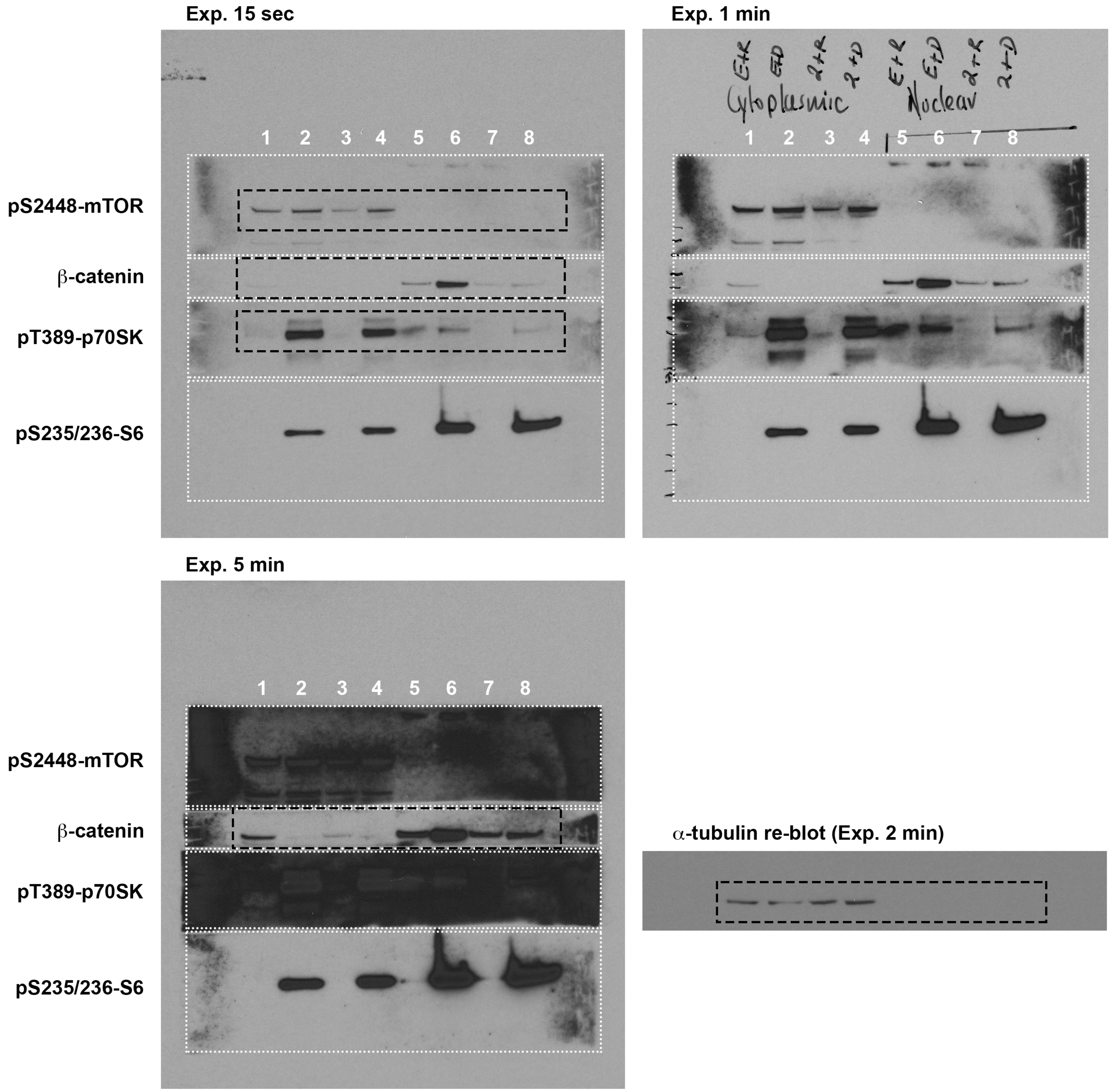

Figure S11. KEGG pathway map (with pathway analysis) and gene expression differences for Wnt signaling between rapamycin-treated ELT3-245 vs ELT3. Genes with negative logFC values (marked in blue) are up-regulated. Genes with positive logFC values (marked in red) are down-regulated.

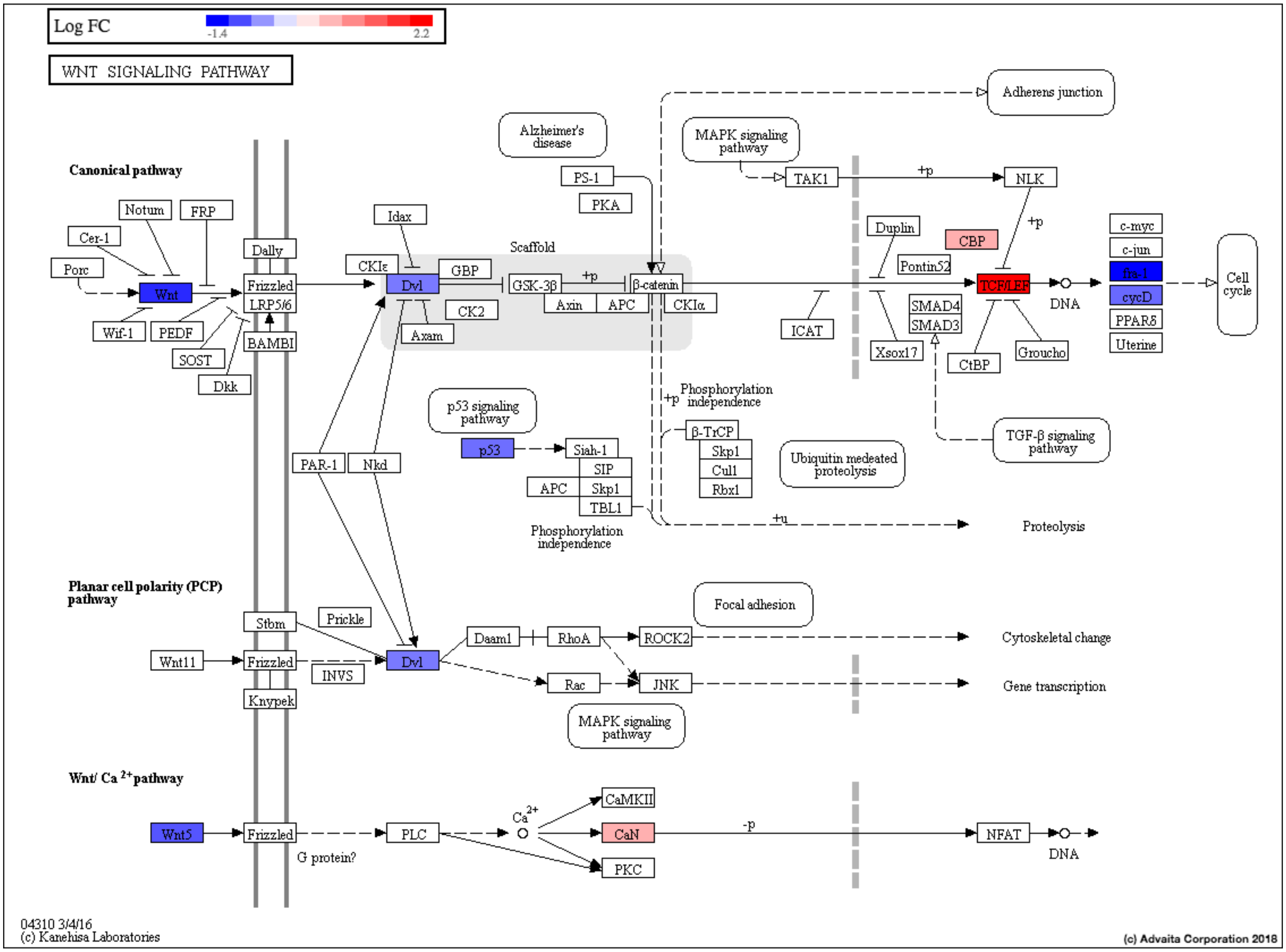

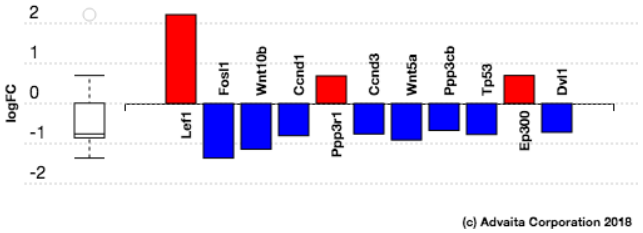

Figure S12. Heat map of matrix metalloproteinase genes that were differentially expressed between rapamycin treated ELT3-245, compared to rapamycin treated ELT3.

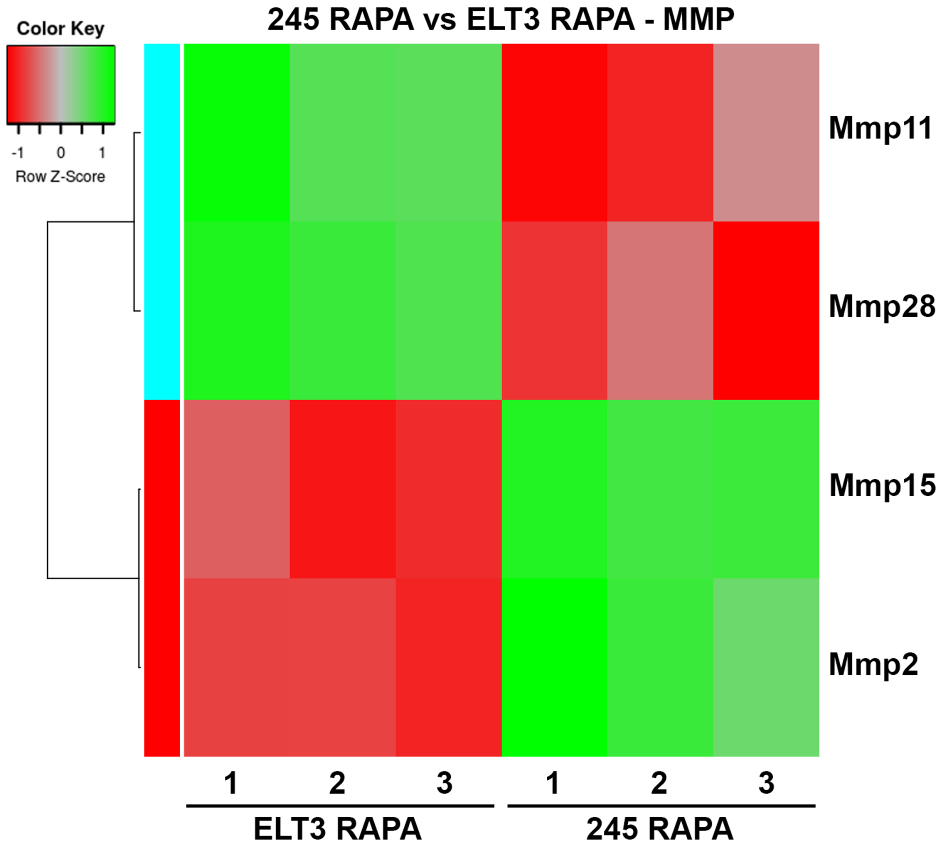

Figure S13. Supplementary images for Fig. 5C. Digital (scanned) images of chemiluminescence signal captured on autoradiography film. Raw (unedited) images are shown. Black dashed-line rectangles correspond to the cropped immunoblot sections shown in Fig. 3E. White dashed lines indicate the membrane boundaries.

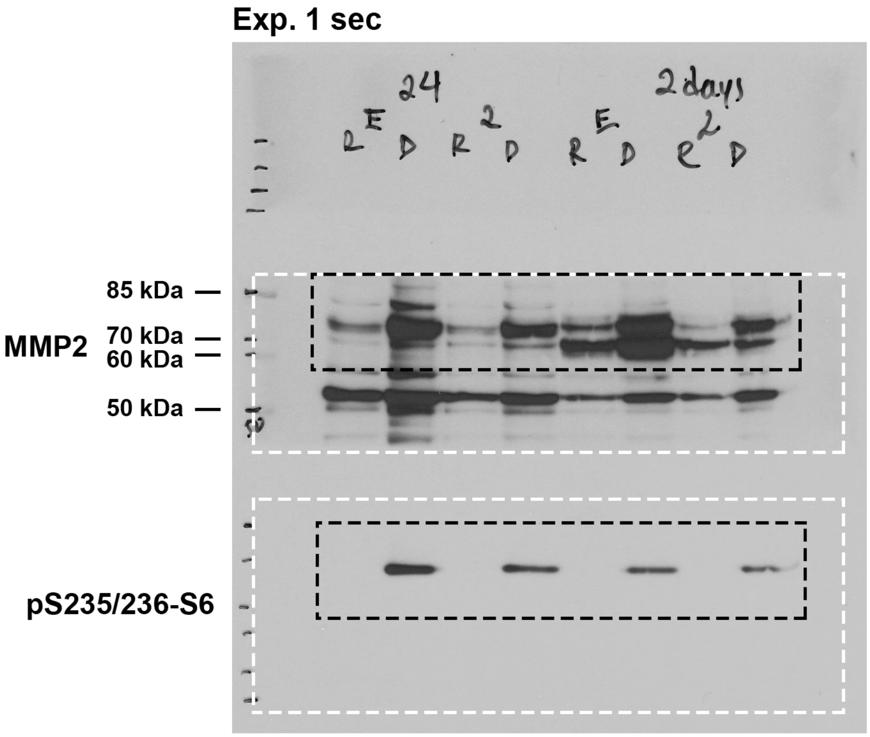

Figure S14. Supplementary images for Fig. 5D. Digital photographs of zymogram gel for MMP2. Raw (unedited) image is shown on the left. Levels-adjusted image is shown on the right. The black dashed-line rectangle corresponds to the cropped gel section shown in Fig. 3F.

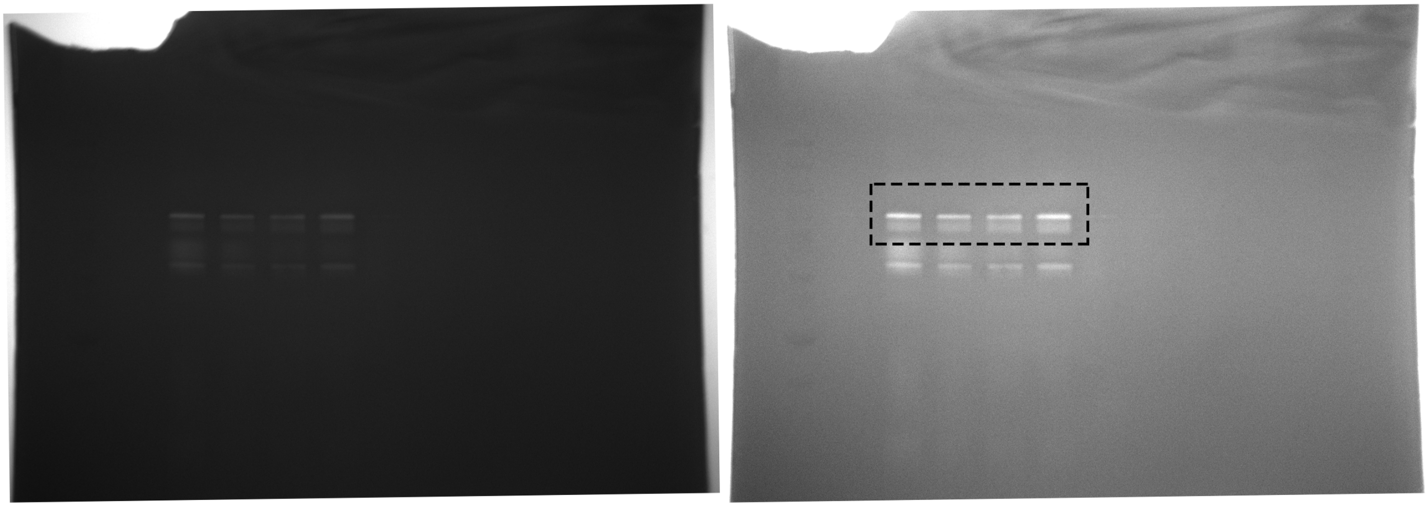

Figure S15. KEGG pathway map (with pathway analysis) and gene expression differences for ErbB signaling between rapamycin-treated ELT3-245 vs ELT3. Genes with negative logFC values (marked in blue) are up-regulated. Genes with positive logFC values (marked in red) are down-regulated.

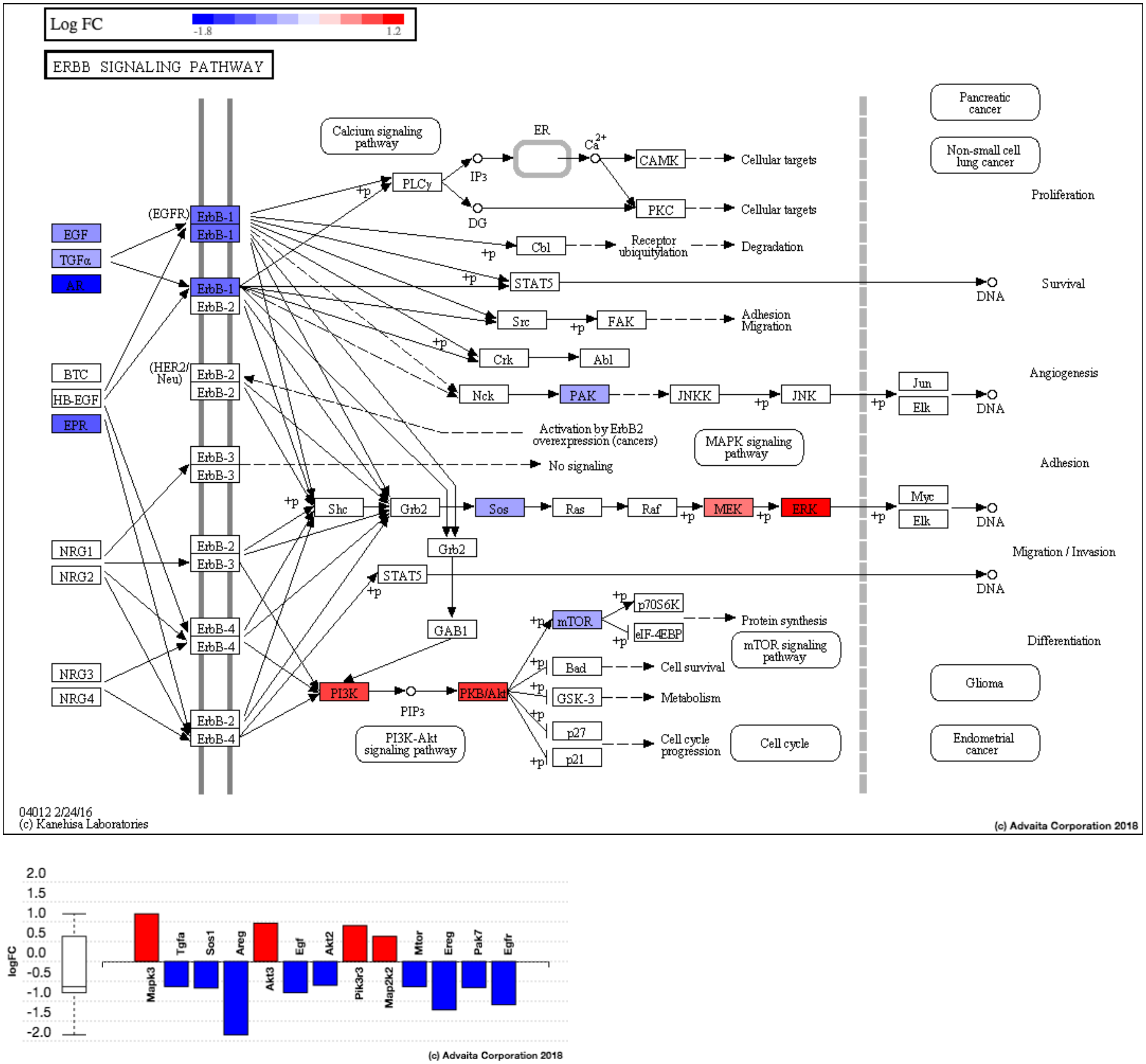

Figure S16. Heat map of ErbB signaling genes that were differentially expressed between rapamycin treated ELT3-245, compared to rapamycin treated ELT3.

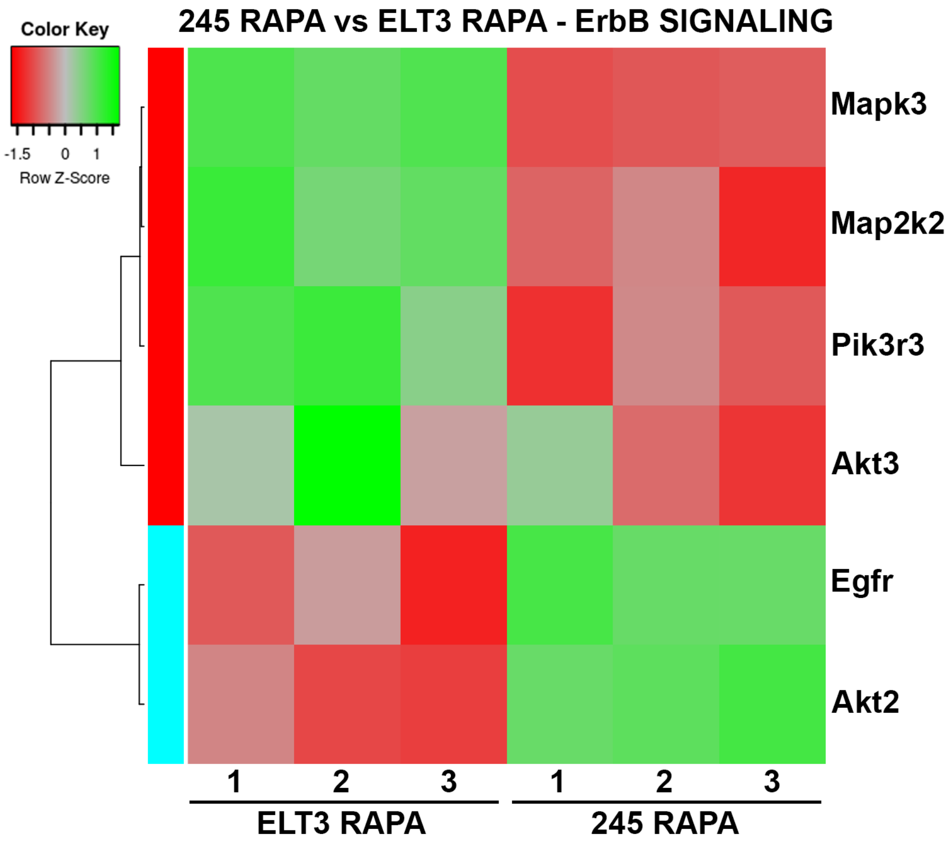

Figure S17. KEGG pathway map (with pathway analysis) and gene expression differences for ECM-receptor interaction signaling between rapamycin-treated ELT3-245 vs ELT3. Genes with negative logFC values (marked in blue) are up-regulated. Genes with positive logFC values (marked in red) are down-regulated.

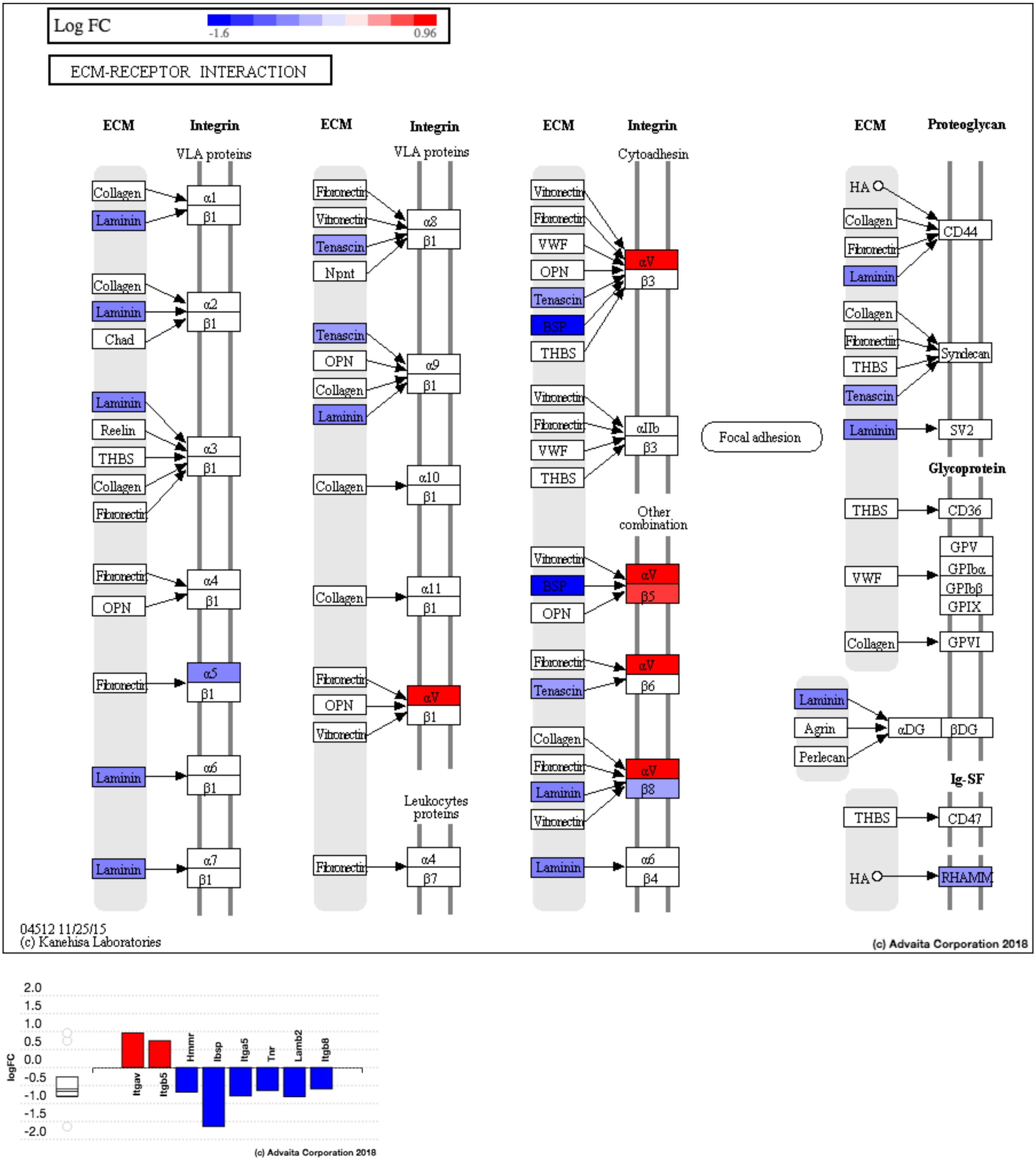

Figure S18. Heat map of integrin genes that were differentially expressed between rapamycin treated ELT3-245, compared to rapamycin treated ELT3.

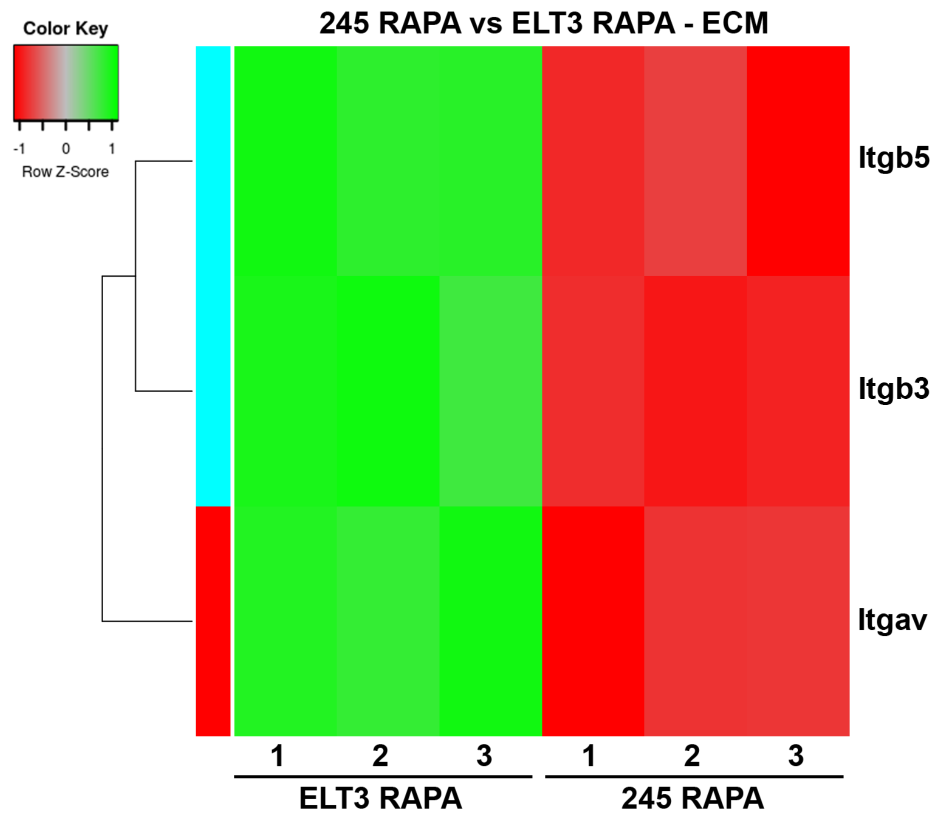

Supplement: Supplementary file 1 — Supplementary Information [file 41598_2019_39418_MOESM1_ESM.pdf]
